# New diterpenes from the marine sponge *Spongionella* sp. overcome drug resistance in prostate cancer by inhibition of P-glycoprotein

**DOI:** 10.1038/s41598-022-17447-x

**Published:** 2022-08-09

**Authors:** Sergey A. Dyshlovoy, Larisa K. Shubina, Tatyana N. Makarieva, Jessica Hauschild, Nadja Strewinsky, Alla G. Guzii, Alexander S. Menshov, Roman S. Popov, Boris B. Grebnev, Tobias Busenbender, Su Jung Oh-Hohenhorst, Tobias Maurer, Derya Tilki, Markus Graefen, Carsten Bokemeyer, Valentin A. Stonik, Gunhild von Amsberg

**Affiliations:** 1 Department of Oncology, Hematology and Bone Marrow Transplantation With Section Pneumology, Hubertus Wald Tumorzentrum–University Cancer Center Hamburg (UCCH), University Medical Center Hamburg-Eppendorf, Hamburg, Germany; 2 Martini-Klinik, Prostate Cancer Center, University Hospital Hamburg-Eppendorf, Hamburg, Germany; 3 Institute of Science-Intensive Technologies and Advanced Materials, Far Eastern Federal University, Vladivostok, Russian Federation; 4 G.B. Elyakov Pacific Institute of Bioorganic Chemistry, Far-East Branch, Russian Academy of Sciences, Vladivostok, Russian Federation; 5 Centre de Recherche du Centre hospitalier de l’Université de Montréal (CRCHUM) Et Institut du Cancer de Montréal, Montreal, QC Canada; 6 Department of Urology, University Hospital Hamburg-Eppendorf, Hamburg, Germany

**Keywords:** Cancer therapy, Urological cancer, Biochemistry, Cancer, Drug discovery, Ocean sciences, Oncology, Chemistry, Biochemistry

## Abstract

Spongian diterpenes are a group of marine natural compounds possessing various biological activities. However, their anticancer activity is still poorly studied and understood. We isolated six spongian diterpenes from the marine sponge *Spongionella* sp*.*, including one new spongionellol A and five previously known molecules. The structures were elucidated using a detailed analysis MS and NMR spectra as well as by comparison with previously reported data. Two of them, namely, spongionellol A and 15,16-dideoxy-15α,17β-dihydroxy-15,17-oxidospongian-16-carboxylate-15,17-diacetate exhibited high activity and selectivity in human prostate cancer cells, including cells resistant to hormonal therapy and docetaxel. The mechanism of action has been identified as caspase-dependent apoptosis. Remarkably, both compounds were able to suppress expression of androgen receptor (AR) and AR-splice variant 7, as well as AR-dependent signaling. The isolated diterpenes effectively inhibited drug efflux mediated by multidrug-resistance protein 1 (MDR1; p-glycoprotein). Of note, a synergistic effect of the compounds with docetaxel, a substrate of p-glycoprotein, suggests resensitization of p-glycoprotein overexpressing cells to standard chemotherapy. In conclusion, the isolated spongian diterpenes possess high activity and selectivity towards prostate cancer cells combined with the ability to inhibit one of the main drug-resistance mechanism. This makes them promising candidates for combinational anticancer therapy.

## Introduction

Spongian diterpenes are a family of marine-derived compounds which possess a parent of 6,6,6,5-tetracyclic ring system. They can be divided into two main groups, specifically, compounds with an intact spongian skeleton (i.e. 6,6,6,5-tetracyclic core), and compounds with an incomplete or rearranged skeletons. The structures of spongian diterpenoids differ in the degree and pattern of oxidation. These compounds are present in various sources including different marine invertebrate species. Of note, the marine sponges belonging to the orders Dictyoceratida and Dendroceratida have been identified as the richest sources of spongian diterpenoids^1^.

A broad range of biological activities, such as antifungal, anti-inflammatory, and antiviral effects among others, have been reported for spongian diterpenes stimulating further efforts on synthesis and modifications of these molecules and their derivatives^1,2^. In contrast, hardly any cytotoxic effects have been demonstrated for the majority of the molecules belonging to this family of natural compounds, including a virtually non-existent effect on malignant cells^1–5^. However, following a chemical modification the newly developed semisynthetic derivates revealed pronounced anticancer activity in vitro^6,7^. Despite of a long time since these molecules have been discovered, their mechanisms of action are still poorly understood^8^. In addition, the knowledge on molecular targets of spongian diterpenes is still limited. Briefly summarized, it is known so far, that their anti-inflammatory activity is linked to the ability to inhibit phospholipase A2, while cytotoxic apoptosis-inducing activity reported for some natural spongian diterpenes in cancer cells can be partially explained by inhibition of DNA polymerase β lyase^9–11^ and suppression of NFκB activity^10^. This is in line with the inhibition of DNA biosynthesis reported for related molecular compounds^12^.

Interesting results have been reported on the activity of several spongian diterpenoids in human prostate cancer (PCa) cell lines. Here, antiproliferative activity was mediated by inhibition of androgen receptor (AR) signaling^13^. The AR is a transcriptional factor, which is activated by binding androgens (e.g. testosterone). Activated AR translocates to the nucleus where it leads to the transcription of various genes, including *KLK3*, which encodes prostate-specific antigen (PSA). Functional AR signaling is essential for survival and progression of PCa cells, especially in the early, hormone-sensitive stage of the tumor development. For this reason, inhibition of the AR pathway has become a key target in the treatment of metastatic PCa^14,15^. Presumably, in non-tumor models the above mentioned spongian diterpenoids inhibited AR activity via binding and therefore blocking of AR due to the structural similarity to testosterone, a natural ligand of AR. Additionally, downregulation of total AR at the protein level was found, which may also have contributed to the inhibition of AR signaling^13^.

Taxane-derived drugs, such as docetaxel and cabazitaxel are frequently used for the treatment of advanced PCa, while the further development of paclitaxel has not been pursued. With time most of the PCa patients develop a resistance to these drugs^16^. Here, a major mechanism of resistance is overexpression of p-glycoprotein (p-gp, multidrug-resistance-protein 1, MDR1), a molecular pump that mediates the elimination of drugs out of the cells, including taxanes^17^. Paclitaxel and docetaxel have high affinity to p-gp, whereas cabazitaxel has been designed to have less propensity to the p-gp-mediated drug excretion and therefore is still beneficial after docetaxel failure^18^. Because it lowers the intracellular concentration of a variety of chemotherapeutic agents, p-glycoprotein is an attractive target in tumor therapy. In fact, effective inhibition of the transporter can lead to resensitization of tumor cells to standard therapies^17^.

In the current study, in continuation of a search for bioactive compounds from the Northwestern Pacific marine invertebrates^19–21^, we investigated the marine sponge *Spongionella* sp., collected in the Sakhalin Gulf (Sea of Okhotsk, Pacific Ocean). We report the isolation of a new as well as previously known compounds identified as spongian diterpenes. These molecules were further investigated in PCa cells, as promising data have been previously reported in this model^13^. Activity, selectivity, and targeting of p-glycoprotein, as one of the major molecule mediating drug-resistance of cancer cells, are described. Our research contributes to the understanding of mechanisms of action of spongian diterpenes and highlights their potential as drug candidates for combinational therapy of cancer.

## Materials and methods

### General procedures for chemical experiments

1D and 2D NMR spectra were recorded on a Bruker Avance III-700, and Bruker Avance III HD-500 spectrometers (Bruker, Ettlingen, Germany). TMS was used as an internal standard. HRESIMS analyses were performed using Bruker maXis Impact II mass spectrometer (Bruker Daltonics, Bremen, Germany). For measurement of the optical rotations a PerkinElmer 343 polarimeter (Waltham, MA, USA) was used, the measures were performed at 20 °C. IR spectra were recorded on a spectrophotometer Equinox 55 (Bruker, Ettlingen, Germany). High-performance liquid chromatography (HPLC) was performed using Shimadzu Instrument with differential refractometer RID-10A (Shimadzu Corporation, Kyoto, Japan) as well as YMC-Pack ODS-A (250 × 10 mm) column (YMC Co., Ltd., Kyoto, Japan). Low-pressure column liquid chromatography was performed using YMC*Gel ODS-A sorbent (YMC Co., Ltd., Kyoto, Japan).

### Animal material

Specimens of *Spongionella* sp*.* were collected in the Gulf of Sakhalin (54°31,6°N; 140°45,0 E) by dredging at 82 m depth on August 2020, and identified by Grebnev B.B. A voucher specimen was deposited under registration number 059-01 in the collection of marine invertebrates of the G.B. Elyakov Pacific Institute of Bioorganic Chemistry.

### Extraction and isolation of compounds

The freshly collected specimens were immediately frozen and stored at − 18 °C until use. Animal material (dry weight 25 g) were crushed and extracted with EtOH (2 × 0.7 L). After evaporation in vacuo the EtOH extract was fractioned by flash column chromatography on YMC*Gel ODS-A (75 μm), eluting with a step gradient of H_2_O–EtOH (60:40–0:100) with monitoring by HPLC. The fractions that eluted with 60% EtOH were further purified by repeated reversed phase HPLC (YMC-ODS-A column (250 × 10 mm), 1.7 mL/min, H_2_O-EtOH, 20:80) to afford, in order of elution, compounds **3** (2.0 mg, 0.008% of dry weight), **2** (3.0 mg, 0.012% of dry weight), **1** (1.5 mg, 0.006% of dry weight), **4** (5.0 mg, 0.02% of dry weight), **5** (3.0 mg, 0.012% of dry weight), and **6** (1.0 mg, 0.004% of dry weight) with retention time (*t*_R_) of 11.0, 12.4, 13.4, 17.0, 19.6, and 29.1 min, respectively. The structures and purity of the individual compounds has been established based on NMR and MS data.

### Compound characterization data

Compound **1**, Spongionellol A: colorless oil;  − 30 (*c* 0.1, CHCl_3_); IR (CHCl_3_) ν_max_ 2992, 1738, 1368 cm^−1^; ^1^H and ^13^C NMR data, Table 1; HRESIMS *m/z* 531.2545 [M+Na]^+^ (calcd. for C_27_H_40_NaO_9_, 531.2565).

**Table 1:** NMR Data for compound **1** in CDCl_3_.

Position	δ_H_ (J in Hz)	δ_C_ type
1α	0.93, td (13.1, 3.5)	38.8, CH_2_
1β	1.67, m	
2α	1.47, m	18.3, CH_2_
2β	1.62, m	
3α	1.17, td (13.2, 4.0)	41.7, CH_2_
3β	1.42, m	
4		32.5, C
5	1.30, dd (13.2, 1.8)	48.2, CH
6α	1.90, m	24.6, CH_2_
6β	1.43, m	
7	5.45, t (3.0)	73.4, CH
8		51.0, C
9	1.72, m	44.5, CH
10		37.9, C
11α	1.71, m	14.8, CH_2_
11β	1.42, m	
12α	1.89, m	19.2, CH_2_
12β	1.73, m	
13	2.78, ddd (13.2, 5.2, 2.5)	37.8, CH
14	2.73, dd (5.2, 1.4)	51.4, CH
15	6.16, s	99.7, CH
16		174.2, C
17	6.62, s	98.8, CH
18	0.77, s	21.2, CH_3_
19	0.76, s	33.2, CH_3_
20	0.85, s	14.2, CH_3_
7-OCO*CH*_*3*_	2.12, s	21.4, CH_3_
15-OCO*CH*_*3*_	2.03, s	21.2, CH_3_
17-OCO*CH*_*3*_	2.13, m	21.3, CH_3_
-O*CH*_3_	3.68, s	52.0, CH_3_
7-O*C*OCH_3_		169.9, C
15-O*C*OCH_3_		169.6, C
17-O*C*OCH_3_		169.4, C

Compound **2**: amorphous solid;  − 40 (*c* 0.2, CHCl_3_), lit.  – 35 [5]; ^1^H and ^13^C NMR data, Figs. S8, S9; HRESIMS *m/z* 415.2088 [M+Na]^+^ (calcd. for C_22_H_32_NaO_6_, 415.2091).

Compound **3**: amorphous solid;  − 20 (*c* 0.2, CHCl_3_), lit.  − 21.7 [5], ^1^H and ^13^C NMR data, Supplementary Figs. S10, S11; HRESIMS *m/z* 373.1980 [M+Na]^+^ (calcd. for C_20_H_30_NaO_5_, 373.1985).

Compound **4**: amorphous solid;  − 26 (*c* 0.1, CHCl_3_), lit.  − 31.8 [6]; ^1^H and ^13^C NMR data, Supplementary Figs. S12, S13; HRESIMS *m/z* 357.2033 [M+Na]^+^ (calcd. for C_20_H_30_NaO_4_, 357.2036).

Compound **5**: colorless oil;  + 37 (*c* 0.2, CHCl_3_), lit. [α]—unpublished; ^1^H and ^13^C NMR data, Supplementary Figs. S14, S15; HRESIMS *m/z* 473.2521 [M+Na]^+^ (calcd. for C_25_H_38_NaO_7_, 473.2510).

Compound **6**: amorphous solid;  − 1 (*c* 0.1, CHCl_3_), lit.  − 3 [10], [α]_578_ − 2.3^22,23^,  + 53 [9]; ^1^H and ^13^C NMR data, Supplementary Figs. S16, S17; HRESIMS *m/z* 327.2283 [M+Na]^+^ (calcd. for C_20_H_32_NaO_2_, 327.2295).

### Reagents and antibodies for biological experiments

Calcein-AM was purchased from BIOZOL (Eching, Germany); tariquidar - from MedChemExpress (Monmouth Junction, NJ, USA); MTT (3-(4,5-dimethylthiazol-2-yl)-2,5-diphenyltetrazolium bromide) - from Sigma (Taufkirchen, Germany); cOmplete™ *EASY*packs protease inhibitors cocktail and PhosphoSTOP™ *EASY*packs - from Roche (Mannheim, Germany); docetaxel and cisplatin - from the Pharmaceutical Department of the University Hospital Hamburg-Eppendorf (Hamburg, Germany). Primary and secondary antibodies used are listed in Supplementary Table S1.

### Cell lines and culture conditions

The following cell lines were used: PC3, DU145, 22Rv1, VCaP and LNCaP (human prostate cancer cells), as well as PNT2 and RWPE-1 (human prostate non-cancer cells) purchased from ATCC (Manassas, VA, USA). MRC-9 (human fibroblast cells) and HEK 293T (human embryonic kidney cells) purchased from ECACC (Salisbury, UK). PC3-DR and DU145-DR (docetaxel-resistant human prostate cancer cells), generated through a long-term cultivation of PC3 and DU145 cells, respectively, in the step-wise increasing concentrations of docetaxel as described previously and were kindly provided by Prof. Z. Culig, Innsbruck Medical University, Innsbruck, Austria^24^. All the cells were recently authenticated by Multiplexion (Heidelberg, Germany). The culture conditions were previously published^25,26^.

### MTT assay

Effect on cell viability (metabolic activity) was examined by MTT assay, which was performed as previously reported^26^. Cells were seeded in 96-well plates (6 × 10^3^ cells/well), incubated overnight, and treated with the indicated concentrations of the drugs in 100 μl/well for 48 h, unless otherwise stated. Then MTT reagent was added to each well, the plates were incubated for 2 h following measurement of the viability.

### Trypan blue staining viability assay

Effect on cell viability (cellular membrane integrity) was examined by trypan blue exclusion assay which was performed as previously reported^26^. Cells were seeded in 6-well plates (200 × 10^3^ cells/well), incubated overnight, and treated with the indicated concentrations of the drugs in 2 mL/well for 48 h. Cell were harvested by trypsination, and analyzed using automatic staining with trypan blue performed by Beckman Coulter Vi-CELL (Beckman Coulter, Krefeld, Germany).

### Analysis of cell cycle progression and DNA fragmentation

The effects DNA fragmentation and cycle progression were analyzed by flow cytometry which was performed as previously reported^27^. Cells were seeded in 12-well plates (100 × 10^3^ cells/well), incubated overnight, and treated with the indicated concentrations of the tested compounds in 1 mL/well for 48 h. Cell were harvested by trypsination, fixed, stained with propidium iodide, and analyzed using FACS Calibur (BD Bioscience, San Jose, CA, USA). The cells containing fragmented DNA were detected as sub-G1 population.

### Detection of apoptotic cells by annexin-V-FITC/PI double staining

Externalization of phosphatidylserine was measured by flow cytometry using annexin-V-FITC and propidium iodide (PI) double staining performed as previously reported^27^. Cells were seeded in 12-well plates (200 × 10^3^ cells/well), incubated overnight and pre-treated with 100 µM of z-VAD(OMe)-fmk for 1 h in 1 mL/well, followed by a co-treatment with the tested drugs for 48 h. Cell were harvested by trypsination, stained, and analyzed using FACS Calibur (BD Bioscience, San Jose, CA, USA).

### Combinational treatment with standard therapies

The effect of the compounds in combination with docetaxel was determined using the Chou-Talalay method^28,29^ performed as described before^26,30^. The cells were co-treated with different concentrations of the drugs for 48 h in 100 µL/well. Cell viability was measured by MTT assay and the data were processed using SynergyFinder 2.0 software (https://synergyfinder.fimm.fi^31^). The analysis was performed as described before^26,30^. On the generated 2D maps deviations between expected and observed effects with positive (red regions) and negative δ-values (green regions) correspond to synergism and antagonism.

### Western blotting

Effect on protein expression was analyzed by Western blotting which was performed as described before^27^. Cells (1 × 10^6^ cells/petri dish) were incubated overnight and treated with the investigated drugs at indicated concentrations for 48 h. Cells were harvested using lysis buffer containing protease and phosphatase inhibitors and the proteins were subjected to further separation and analysis. For detection of multiple proteins on the same membrane, the membranes were cut following the transfer according to the molecular weight of the proteins of interest. The membranes were incubated in the correspondent primary and secondary antibodies and further developed according to the manufacturer’s protocols. If multiple proteins were consequently detected on the same membrane piece the stripping step was used. In each experiment the proteins of interest and the loading control were detected on the same membrane. The antibodies used for detection are listed in Supplementary Table S1. The original full-size blots are represented in Supplementary Figs. S18–S20.

### Analysis of p-glycoprotein activity

The p-gp overexpressing PC3-DR cells (6 × 10^3^ cells/well) were seeded in black clear bottomed 96-well plates in docetaxel-free culture medium (100 µL/well) and the plates were incubated overnight. The media was exchanged with DPBS (50 µL/well) containing investigated drugs at the indicated concentrations and the plates were incubated for 30 min. Then 50 µL/well of calcein-AM solution in DPBS (1 µM) were added, the plates were incubated for another 15 min and the green fluorescence was measured using Infinite F200PRO reader (TECAN, Männedorf, Switzerland). The values were normalized to the possible background fluorescence of the drugs’ solutions, which was measured in the same conditions but without addition of calcein-AM dye. For viability measurement, 50 µL/well of MTT solution in drug-free medium were added instead of calcein-AM solution and the viability was measured followed by 2 h incubation as described for MTT assay.

### Data and statistical analysis

All the experiments were performed in triplicates (*n* = 3, biological replicates). The cells treated with the solvent alone were used as a control. Statistical analyses and calculations of IC_50_s were performed using GraphPad Prism v.9.1.1 software (GraphPad Software, San Diego, CA, USA). Data are presented as mean ± standard deviation (SD). For groups comparison the one-way ANOVA followed by Dunnett's post-hoc tests were used. Statistically significance of the difference is indicated as “*” (if *p* < 0.05).

## Results and discussion

### Isolation and characterization of the active compounds from marine sponge *Spongionella* sp

**Figure 1:**
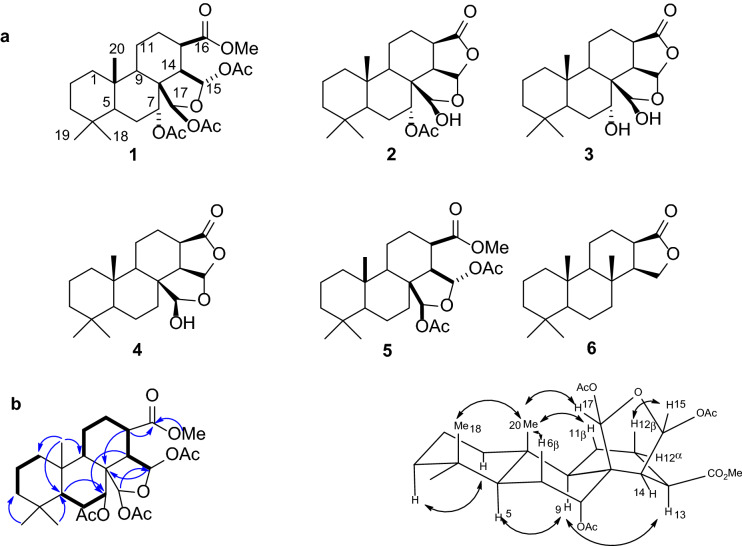
Diterpenes from marine sponge *Spongionella* sp. (**a**), The structures of compounds **1**–**6**. (**b**), Key COSY (), HMBC () and NOESY () correlations for the new compound **1.**

The EtOH extract of the sponge *Spongionella* sp. was concentrated and the obtained residue was fractionated by flash chromatography on a YMC*Gel ODS A column. Further separation using reversed-phase HPLC resulted in the isolation of one new spongian diterpene (**1**, Fig. 1a), named spongionellol A and five known spongian diterpenes (**2**–**6**, Fig. 1a). The structures of the known compounds were determined as 7α,17β,-dihydroxy-15,17-oxidospongian-16-one 7 acetate, (aplyroseol-2, **2**)^32,33^, 7α,17β,-dihydroxy-15,17-oxidospongian-16-one (**3**)^33^, 17β,-dihydroxy-15,17-oxidospongian-16-one (dendrillol-1, **4**)^34,35^, methyl 15,16-dideoxy-15α,17β-dihydroxy-15,17-oxidospongian-16-carboxylate 15,17-diacetate (**5**)^35^, and spongian-16-one (**6**)^36^ on the basis of detailed spectroscopic analysis and comparison with reported data.

Diterpene **1** was isolated as a colorless oil and its molecular formula was determined to be C_27_H_40_O_9_ from the [M + Na]^+^ ion peak at *m/z* 531.2545 in the positive HRESIMS. The ^1^H NMR and ^13^C NMR (Table 1) spectra showed the presence of three methyl groups (δ_H_ 0.85, 0.77, 0.76; δ_C_ 14.2, 21.2, 33.2), three acetate methyls (δ_H_ 2.13, 2.12, 2.03; δ_C_ 21.3, 21.4, 21.2) a carboxymethyl (δ_H_ 3.68; δ_C_ 52.0), and two acetal groups (δ_H_ 6.62, 6.16; δ_C_ 98.8, 99.7), confirmed by HSQC, HMBC and COSY experiments (Fig. 1b). These data closely resembled that of **5**^35^, except for an additional acetate group signals. The HMBC correlations from the oxygenated methine proton H-7 (δ_H_ 5.45) and acetate methyl protons (δ_H_ 2.12) to carbonyl carbon (δ_C_ 169.9) indicated that the additional acetate group was attached to C-7 (Fig. 1b).

The relative configuration of **1** was established from the coupling constants and NOESY experiments (Fig. 1b). The NOE correlations H-6β, H_3_-18 and H_3_-20 implied that H- 6β, H_3_-18, and H_3_-20 were on the same β side of the molecule. The additional correlations between H_3_-20 and H-11β, H-17 showed that H-11β, and H-17 were also on the β side. The large coupling constant (13.2 Hz) between H-5 and H-6β indicated that both protons were in axial-axial orientation and H-5 was on the α side of the molecule. The small axial-equatorial (3.0 Hz) coupling constant between H-6 and H-7 confirmed a β orientation for H-7. The axial-axial coupling constant with H-2β (13.1 Hz) and axial-equatorial coupling constant with 2α (3.5 Hz) suggested H-1α axial orientation. The NOE correlation between H-1α and H-3α indicated that 3α was also in an axial orientation and on the α side of the molecule. These data indicated that rings A and B are in a chair conformations and that ring E is in β orientation to the rest of the molecule (Fig. 1b). NOE correlations between H-9 and H-13 indicated that H-13 is on the α face of the molecule and ring C is in a boat conformation. This conformation was confirmed by a large axial-axial coupling constant (13.2 Hz) between H-13 and H-12β, a smaller coupling constant (5.2 Hz) between H-13 and H-14 as well as a small (1.4 Hz) W-coupling between H-12α and H-14. The proposed configuration of the tetracyclic system for **1** is consistent with that previously reported for D-ring-opened spongian diterpenes aplyroseols 8–12^36^.

### Activity and selectivity in prostate cancer cells

Next, anticancer potential of the isolated compounds was evaluated. Previously, for related marine derived substances a promising antiproliferative activity has been demonstrated in hormone-sensitive LNCaP cells. Interestingly, it was previously reported that the spongian diterpenoids inhibited AR activity by a mechanism that involved competing with androgen ligands for AR ligand binding domain (LBD) as well as blocking essential N-terminal/C-terminal LBD interactions required for androgen-induced AR transcriptional activity^13^. We therefore assessed the cytotoxic potential of our substances using a panel of seven human prostate cancer cell lines, bearing different levels of treatment resistance. This panel included AR-negative PC3 and DU145 cells which are known to be resistant to various hormonal and standard chemotherapeutics, docetaxel-resistant PC3-DR and DU145-DR cells (derived from PC3 cells and DU145 cells, respectively), AR-FL- (androgen receptor full length) and AR-V7-positive (androgen receptor splice variant V7) hormone-resistant 22Rv1 and VCaP cells, as well as AR-FL-positive hormone-sensitive LNCaP cells. Additionally, to estimate the selectivity to cancer cells, four human normal (non-cancer) cell lines were examined (prostate non-cancer PNT2 and RWPE-1 cells, human embryonic kidney HEK 293 T cells, and human fibroblasts MRC-9). In these experiments, most of the isolated compounds exhibited moderate to strong cytotoxic effects on PCa cells. D-ring-opened compounds **1** and **5** were identified to be most active with IC_50_ of low micromolar or sub-micromolar concentrations (Table 2). Remarkably, these compounds revealed pronounced activity in PC3 and DU145 cells, which are known to be resistant to various hormonal and standard chemotherapeutics (Table 2). Moreover, the diterpenes were active in PC3-DR and DU145-DR cells, that exhibit a strong resistance to docetaxel. Finally, a direct comparison of the cytotoxic activity in cancer versus non-cancer cells indicated compounds **1** and **5** to be the most selective towards PCa cells among the isolated molecules, having selectivity indices (SI) of 5.2 and 1.2, respectively (Table 2). Of note, cisplatin which was used as a reference drug, exhibited a SI = 0.94, indicating a pronounced toxicity in non-cancerous cells which is in line with the side effects of cisplatin-based therapy known from the clinic. Interestingly, modification of the ring D seems to be important for the biological activity of the isolated spongian diterpenes, and the opening of this ring results in increase of both cytotoxicity and selectivity.

**Table 2:** Cytotoxic activity and selectivity of the isolated compounds. IC_50_s were determined using MTT assay after 48 h of treatment.

Compound	IC_50_ [µM]											Mean IC_50_, cancer cells [µM]	Mean IC_50_, non-cancer cells [µM]	Selectivity Index (SI)
	Prostate cancer cells							Non-cancer cells						
	PC3	PC3-DR	DU145	DU145-DR	22Rv1	VCaP	LNCaP	PNT2	RWPE-1	HEK293	MRC-9			
1	0.964 ± 0.11	1.23 ± 0.23	0.936 ± 0.39	1.53 ± 0.16	2.64 ± 0.74	1.3 ± 0.2	1.02 ± 0.57	2.21 ± 0.64	1.14 ± 0.35	22.9 ± 4.4	2.19 ± 0.84	1.37	7.11	5.18
2	35.1 ± 5.9	34.8 ± 3.5	27.9 ± 3.9	36.28 ± 8	20.9 ± 3.9	40.2 ± 7.5	8.1 ± 0.7	7.5 ± 0.6	1.8 ± 0.6	2.8 ± 1.1	8.4 ± 1.7	34.8	25.1	0.72
3	53.3 ± 9.2	84.3 ± 8.3	77.9 ± 5.3	62.5 ± 10.2	40.4 ± 7.2	77.6 ± 8.1	87.6 ± 4.5	34.1 ± 11.9	46.7 ± 11.0	27.6 ± 7.9	54.6 ± 9.0	69.1	40.7	0.59
4	30.9 ± 4.3	31.9 ± 8.7	25.4 ± 9.8	26.3 ± 8.1	22.6 ± 3.2	32.1 ± 9.9	31.7 ± 5.5	13.8 ± 7.5	23.4 ± 5.7	12.5 ± 2.7	33.3 ± 4.1	28.7	20.8	0.72
5	2.51 ± 0.93	3.63 ± 0.69	1.52 ± 0.87	1.92 ± 0.16	3.22 ± 0.70	2.51 ± 1.50	1.82 ± 0.85	1.59 ± 0.66	2.72 ± 0.82	4.46 ± 0.46	3.13 ± 0.54	2.45	2.98	1.22
6	> 100	> 100	> 100	> 100	51.8 ± 9.0	> 100	> 100	> 100	> 100	69.5 ± 8.4	> 100	196	93.3	0.48
Cisplatin	34.6 ± 8.7	4.71 ± 0.86	11.7 ± 3.9	2.29 ± 0.61	0.99 ± 0.65	5.02 ± 1.90	2.75 ± 0.54	9.41 ± 3.2	9.02 ± 2.47	6.44 ± 2.1	6.75 ± 0.48	8.40	7.91	0.94

The values are represented as mean ± SD. Selectivity index (SI) was calculated as [mean IC_50_ in non-cancer cells]/[mean IC_50_ in cancer cells]. Cisplatin was used as a reference drug.

We further evaluated the two most active and selective substances—i.e. new compound **1** and previously known **5**. Consequently, as a model for the following experiments we selected the human PCa 22Rv1 cells, which are known to express AR-FL and AR-V7. These cells are known to be resistant to AR targeted therapy due to the presence of AR-V7. However, at the same time, unlike AR-negative PC3 and DU145 cells, they can be used to monitor the effects on AR signaling because of the presence of the AR-FL receptor and the constantly activated AR-V7.

**Figure 2:**
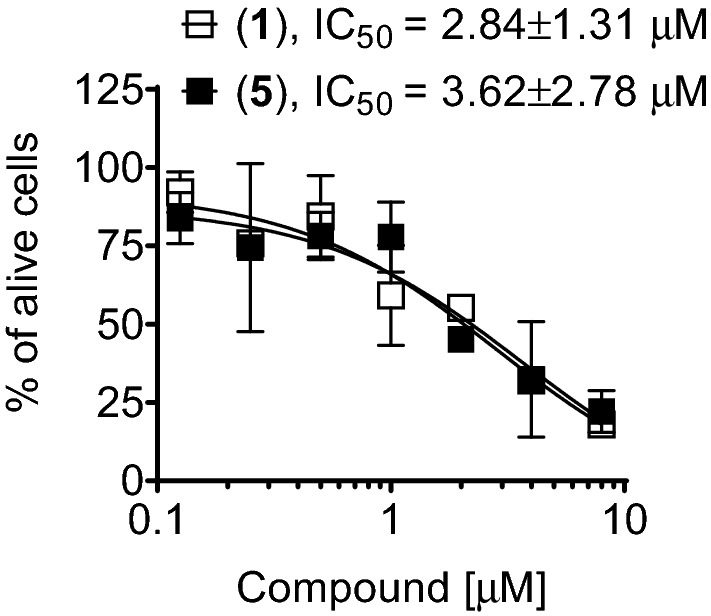
Activity of compounds in 22Rv1 cells determined using trypan blue staining assay. Cells were treated for 48 h. The IC_50_s are represented as mean ± SD.

In order to reveal a possible specific effect on cellular metabolism, we compared the activity assessed by MTT assay with the activity determined by the trypan blue staining assay. MTT assays assess metabolically active cells (having active mitochondria capable of metabolization of MTT to formazan), whereas trypan blue staining detects cellular membrane integrity (the cells having disrupted membrane are stained with trypan blue and are assumed to be dead). Of note, the IC_50_s determined with both MTT and trypan blue staining assays were comparable (Table 2, Fig. 2), suggesting no early specific effect on cellular metabolism, but rather simultaneous targeting of mitochondrial activity and cellular membrane disruption.

### Induction of caspase-dependent apoptosis of cancer cells

**Figure 3:**
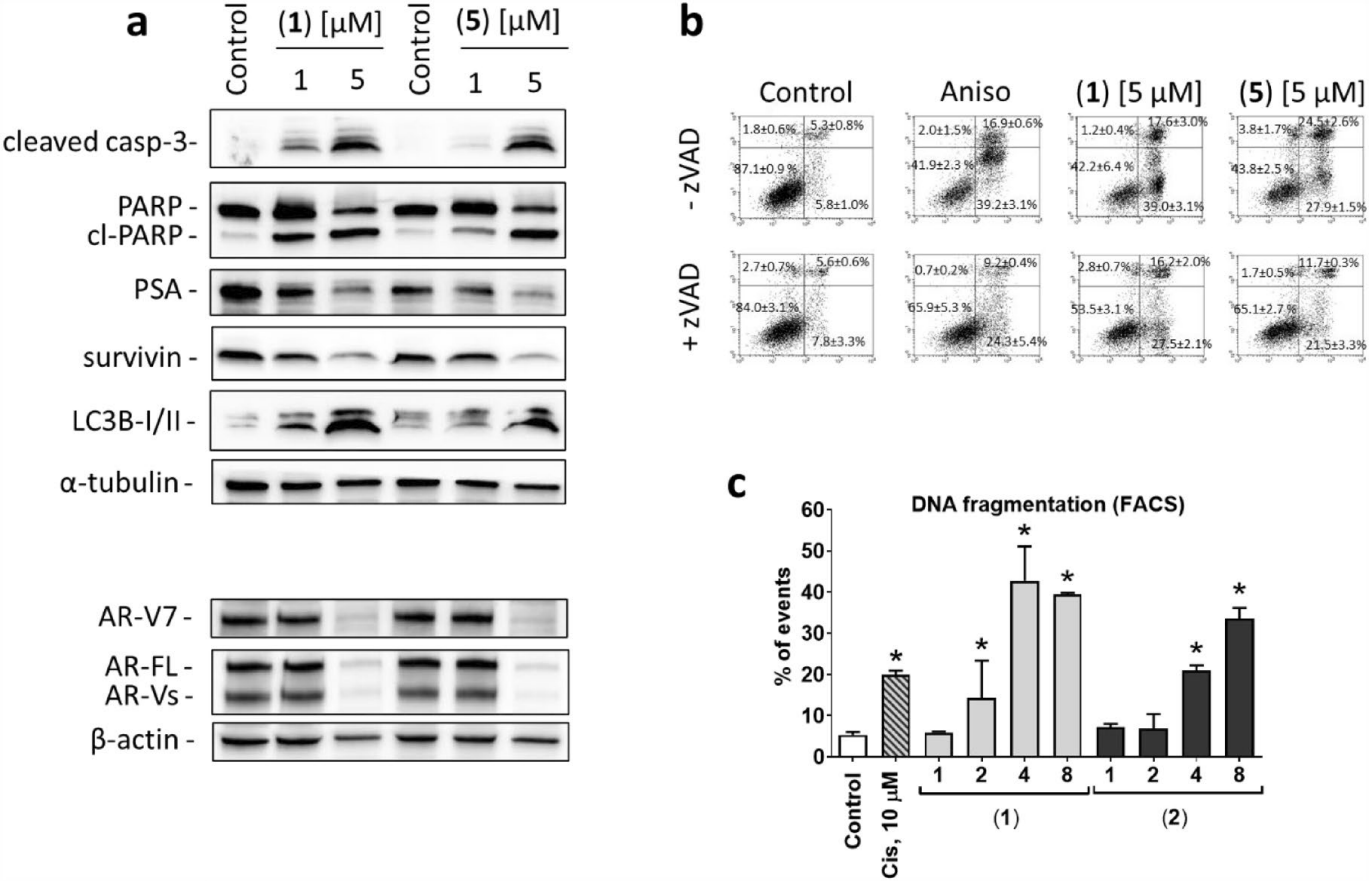
Proapoptotic activity of the isolated diterpenes **1** and **5** in 22Rv1 cells. (**a**), Analysis of protein expression by Western blotting. The original full-size blots are represented in Supplementary Fig. S18. (**b**), Flow cytometry analysis of phosphatidylserine externalization using annexin-V/PI double staining. Cells were pretreated with 100 µM of pan-caspase inhibitor z-VAD(OMe)-fmk (+zVAD) or with the vehicle (-zVAD) for 1 h, and then co-treated with the drugs for another 48 h. Anisomycin (Aniso, 10 µM) was used as a positive control. (**c**), Flow cytometry analysis of DNA fragmentation in the cells treated with the investigated compounds for 48 h. Cisplatin (Cis, 10 µM) was used as a positive control. *p < 0.05, one-way ANOVA test.

To investigate the mode of cytotoxic action of the isolated compounds we examined the effects on apoptosis induction using Western blotting-based analysis of several known pro- and anti-apoptotic proteins (Fig. 3a). Classical apoptosis is characterized by the consequent activation of inducer and effector caspases, followed by phosphatidylserine externalization and DNA fragmentation, which ultimately results in the cellular death. In line with this, a pronounced cleavage of caspase-3 (a protease which is activated via its cleavage and further leads to apoptosis) and PARP (one of the critical enzymes involved in DNA repair) detected in the cells treated with cytotoxic concentrations of both **1** and **5** suggested the induction of caspase-dependent apoptosis which results in the lethal event of DNA fragmentation. To further validate this finding, we used an annexin-V/PI double staining to detect the externalization of phosphatidylserine, which has been recognized as an early apoptotic event. Indeed, the 22Rv1 cells exposed to the drugs exhibited a dose-dependent phosphatidylserine externalization (annexin-V^+^/PI^–^) (Fig. 3b). This effect could be inhibited by pre-treatment with z-VAD(OMe)-fmk, an established pan-caspase inhibitor, which indicates a caspase-dependent nature of cancer cell apoptosis induced by **1** and **5** (Fig. 3b). Finally, a dose-dependent induction of DNA fragmentation was found using PI staining of DNA combined with a flow cytometry analysis (Fig. 3c).

Additionally, we observed a downregulation of the antiapoptotic protein survivin, which may also contribute to the induction of cancer cell death by the investigated compounds (Fig. 3a). At the same time, upregulation of the autophagy marker LC3B-I/II was detected (Fig. 3a). An upregulation of this protein suggests an increased number of autophagosomes. However, this can indicate both, activation or inhibition of autophagy^37^. In most malignancies, cytotoxic agents induce cytoprotective autophagy, a mechanism that protects malignant cells and helps to overcome unfavorable conditions. In contrast, some drugs induce cancer cell death via autophagy mediated cell death^38^. Therefore, the precise effect of spongian diterpenes **1** and **5** on autophagy is still to be clarified.

AR signaling plays an essential role in growth and development of normal and malignant prostate cells. Targeted inhibition of this pathway suppresses the growth of PCa and therefore, such drugs are of particular interest^15^. However, resistance inevitably develops during the course of the treatment. Among others, AR-V7 has been identified as a clinically relevant mechanism of resistance. AR-V7 lacks the C-terminal LBD and is permanently auto-activated. 22Rv1 cells are known to express both, AR-FL and AR-V7. Remarkably, both diterpenes **1** and **5** downregulated AR-FL and AR-V7 in 22Rv1 cells suggesting a suppression of AR-signaling (Fig. 3a). This was confirmed by PSA reduction secondary to drug exposure (Fig. 3a), a known downstream target of AR-FL/AR-V7 signaling. Thus, the isolated diterpenes are able to inhibit AR-signaling in PCa cells presumably by inducing AR-FL and AR-V7 degradation. This is in line with previous findings, which described a suppressive effect of related spongian diterpenes on AR expression and signaling^13^. However, it has not yet been reported that this effect can also be achieved for AR-V7.

### Isolated compounds inhibit p-glycoprotein activity and synergize with docetaxel and cabazitaxel

Another very important observation was that compounds **1**–**6** were equally active in docetaxel-sensitive PC3 and DU145 cells as well as in docetaxel-resistant PC3-DR and DU145-DR cells (Table 2). This is of particular significance since PC3-DR and DU145-DR cells are 40–50-fold more resistant to standard therapy with docetaxel compared to the parental cell line^25^. These drug-resistant cells have been generated via long-term treatment of PC3 or DU145 cells with sub-lethal concentrations of docetaxel^24^. One of the main mechanisms of docetaxel resistance is the overexpression of p-glycoprotein (p-gp, MDR1), a molecular pump that mediates the elimination of chemotherapeutic agents, e.g. docetaxel from inside the cells. Therefore, an overexpression of p-gp results in increased IC_50_ of docetaxel^39^.

**Figure 4:**
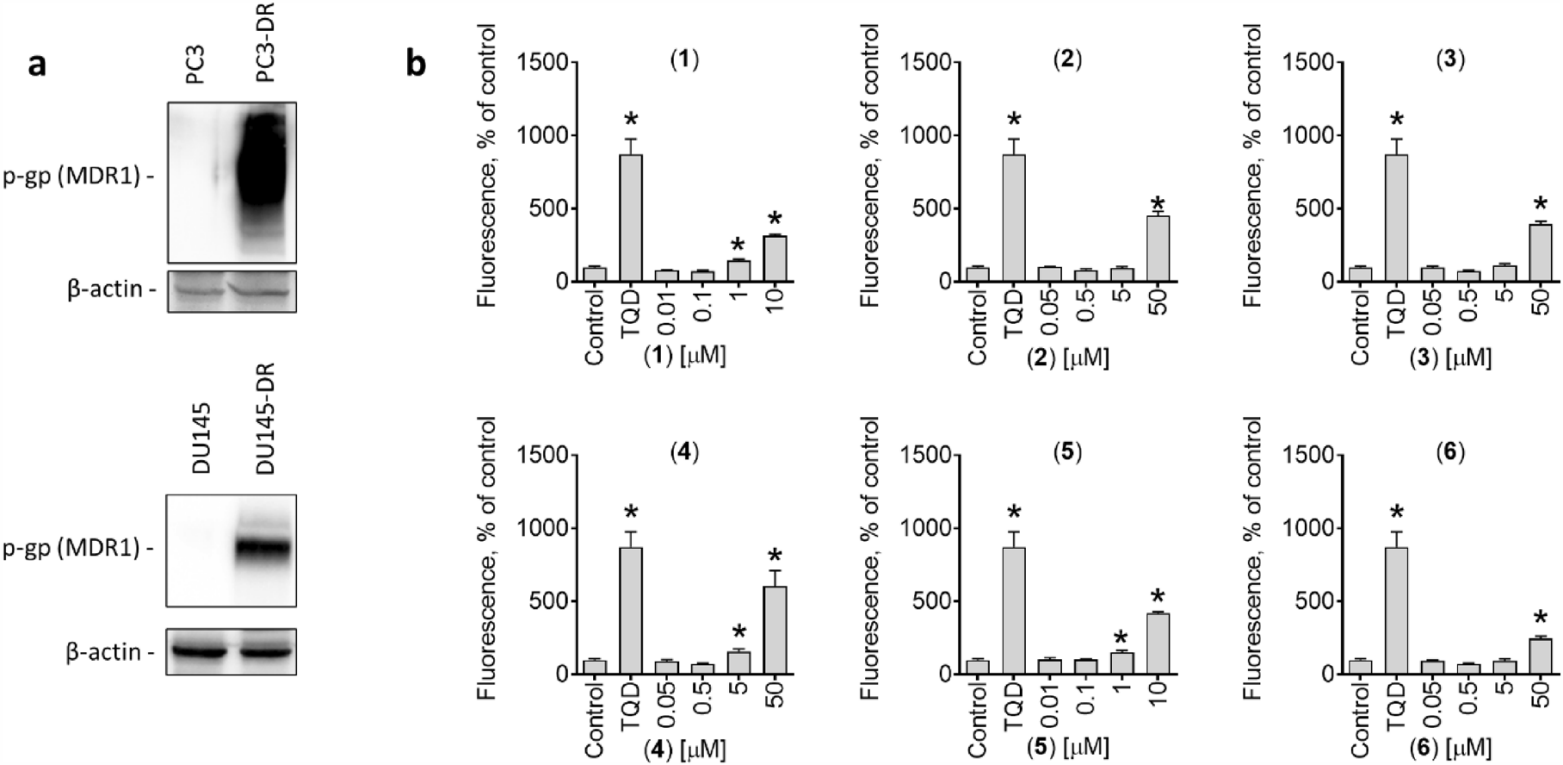
Effect on activity of p-glycoprotein (p-gp). (**a**), Western blotting analysis of p-gp expression in PC3, PC3-DR, as well as in DU145 and DU145-DR cells. The original full-size blots are represented in Supplementary Figure S19. (**b**), Green fluorescence measured in PC3-DR cells pre-treated with investigated compounds for 30 min and then further incubated with calcein-AM for 15 min. Tariquidar (TQD, 50 nM) was used as a positive control. *p < 0.05, one-way ANOVA test.

We found that p-gp is indeed overexpressed in both PC3-DR and DU145-DR cells, which explains the detected resistance to docetaxel (Fig. 4a). At the same time, this indicates that the isolated diterpenes are not a substrate of this pump protein, as no differences of their cytotoxic activity were detected between PC3 and PC3-DR cells, or between DU145 and DU145-DR cells (Table 2). Next, we examined the effects of the isolated compounds on the activity of p-gp using a calcein-AM-exclusion assay. For this assay, the PC3-DR cells expressing high p-gp level have been selected as the main model. Calcein-AM (calcein acetoxymethyl ester) is a non-fluorescent dye which passively diffuses into cells. In metabolic active cells, calcein-AM is further hydrolyzed by cytosolic esterases into its green fluorescent form (calcein) which can further be detected using fluorescent methods. Of note, both calcein and calcein-AM are substrates of p-gp. Thus, both compounds are rapidly excreted out of cells overexpressing p-gp. Consequently, in these cells a significant reduction of green fluorescence can be observed in comparison with the cells with non-overexpressed p-gp. The application of p-gp inhibitors, such as tariquidar (TQD), blocks its activity and results in increased fluorescence due to calcein accumulation. Therefore, it serves as an indicator for p-gp activity and allows to screen for potential inhibitors. A pre-treatment of the PC3-DR cells with the isolated compounds for 30 min followed by incubation with calcein-AM resulted in significant increase in green fluorescence of the cells, indicating treatment-induced reduction of p-gp activity (Fig. 4b).

**Figure 5:**
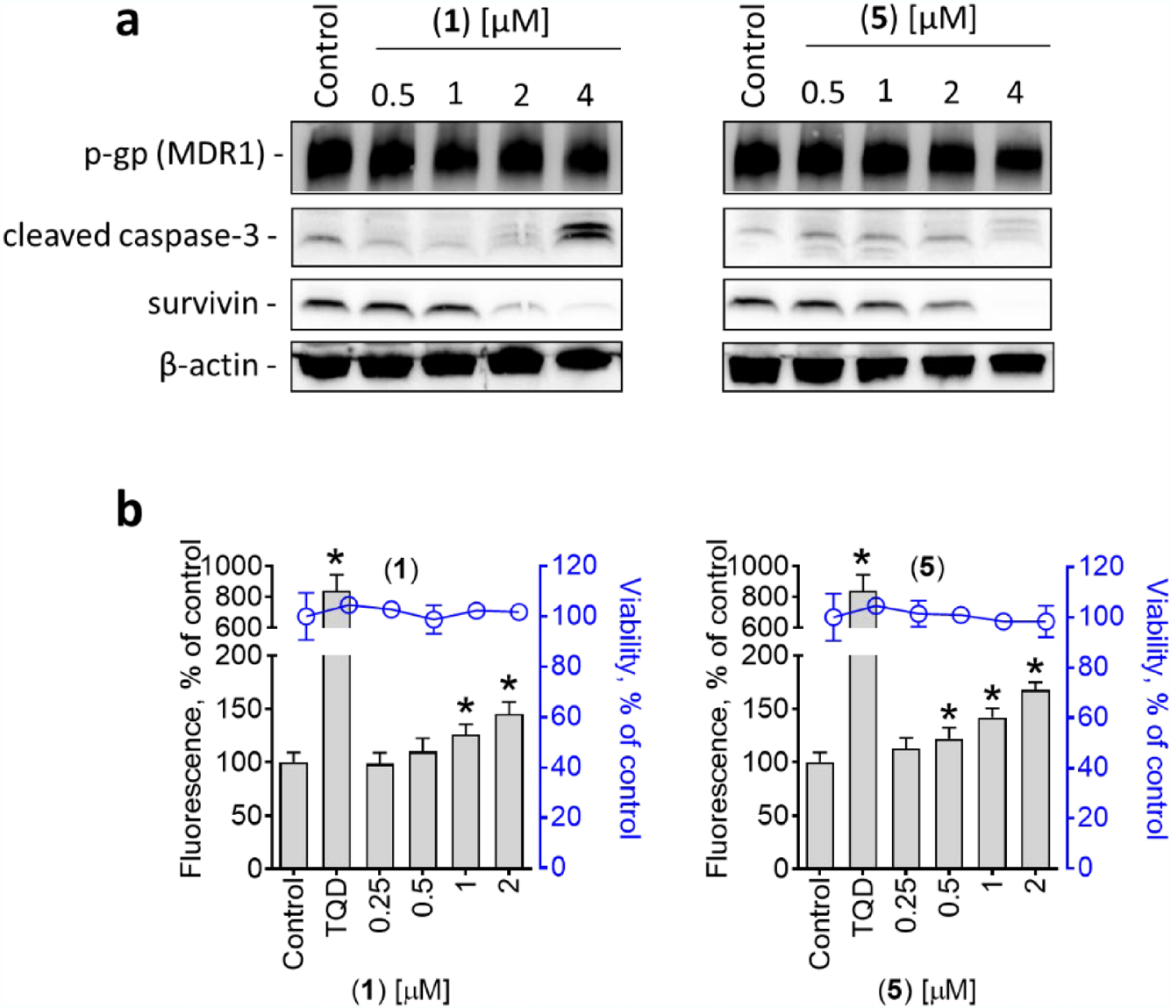
Effect on p-gp expression and viability of PC3-DR cells. (**a**), Western blotting analysis of protein expression in PC3-DR cells treated with the indicated concentration of the compounds for 48 h. The original full-size blots are represented in Supplementary Figure S20. (**b**), Effect on p-gp activity (grey bars) and viability (blue dots) of PC3-DR cells following 30 min exposure to the investigated compounds. p-gp activity was measured using a calcein-AM assay. Cellular viability was measured using MTT assay. Tariquidar (TQD, 50 nM) was used as a positive control. *p < 0.05, one-way ANOVA test.

The reduction of p-gp activity, however, may also be a result of its degradation or cell death related events. To exclude this, we evaluated the effect of the compounds on p-gp expression in PC3-DR cells. Up to 4 µM, no significant changes of p-gp expression were detected. At the same time and in line with the above reported for 22Rv1 cells data, a dose-dependent activation of caspase-3 and a downregulation of survivin were observed in PC3-DR cells (Fig. 5a). Next, we evaluated the viability of the PC3-DR cells exposed to the drugs and aligned it with fluorescence of the cells incubated with calcein-AM measured at the same experimental conditions (Fig. 5b). Remarkably, cellular fluorescence significantly increased, while no changes of viability were detected (Fig. 5b). Based on these observations it can be assumed that the effect described above on calcein accumulation neither results from a p-gp downregulation, nor from any cell death-related processes.

**Figure 6:**
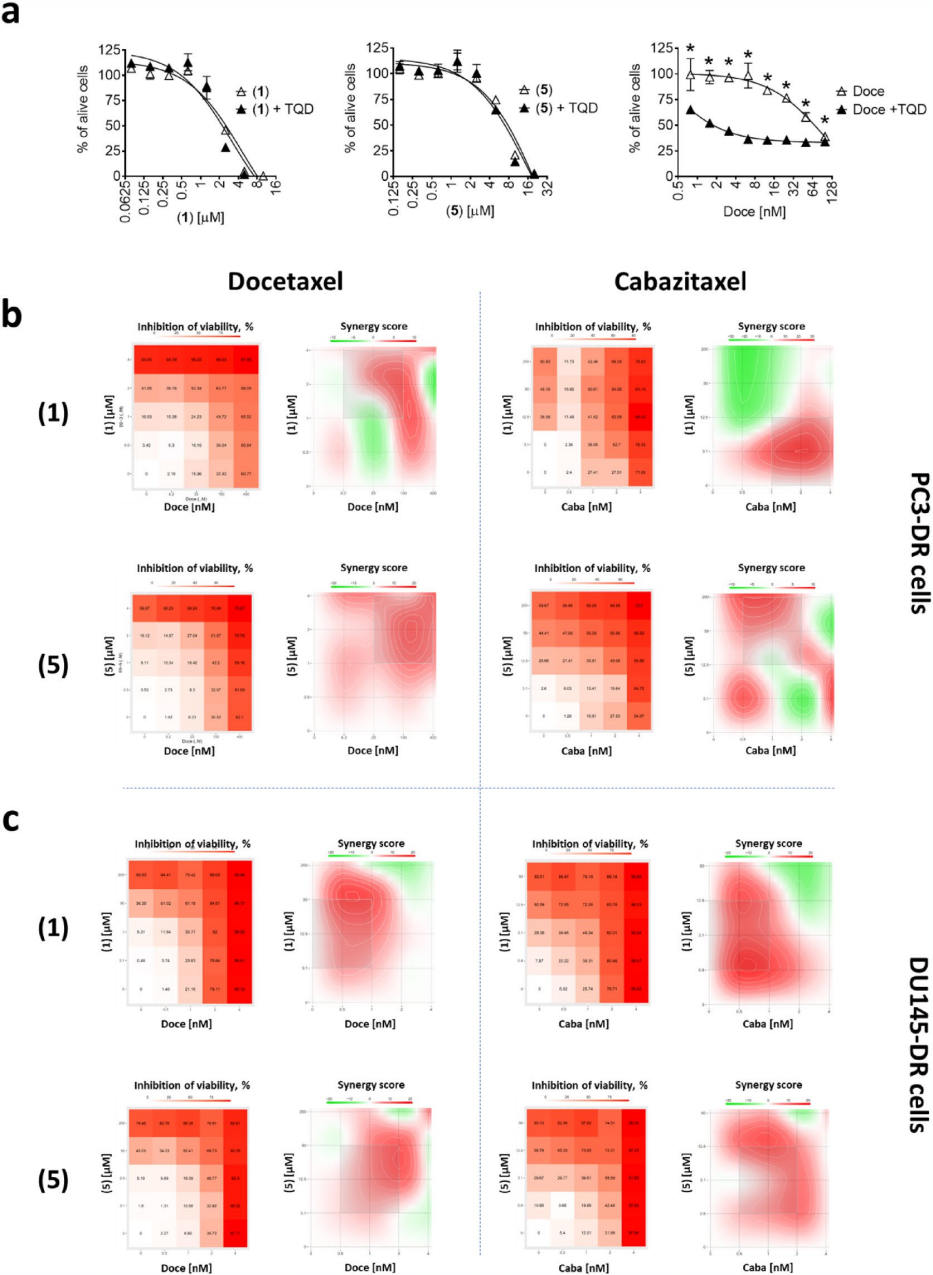
Effects of the drug combinations on viability of PC3-DR and DU145-DR cells. (**a**), PC3-DR cells were pretreated with tariquidar (TQD, 50 nM) for 30 min and then co-treated with the indicated drugs for 48 h. (**b**,**c**), PC3-DR (**b**) and DU145-DR (**c**) cells were co-treated with docetaxel (Doce) or cabazitaxel (Caba) in combination with diterpenes **1** or **5** at the indicated concentrations. Cellular viability was measured using MTT assay. The cytotoxicity heat-maps (viability inhibition) as well as synergistic/additive/antagonistic effects of drug combinations were analyzed and visualized using SynergyFinder 2.0 software (https://synergyfinder.fimm.fi/) using a ZIP algorithm (**b**,**c**). Red areas indicate synergistic effects of the drug combinations. *p < 0.05, one-way ANOVA test.

Competitive inhibitors bind to the same binding site of receptor as the ligand itself, whereas noncompetitive inhibitors suppress the function of a protein by alternative pathways. Docetaxel and some other taxanes can be considered as both, p-gp transport substrates and competitive p-gp transport inhibitors. Thus, docetaxel can bind to p-gp (and is further evacuated from the cell) and therefore competitively inhibits p-gp-mediated calcein excretion resulting in increased intracellular fluorescence. In order to discriminate whether or not compounds **1** and **5** competitively inhibit p-gp due to their p-gp substrate-like activity, we assessed their cytotoxicity in PC3-DR cells in combination with tariquidar (TQD), a selective noncompetitive p-gp inhibitor (Fig. 6a)^40^. In these experiments, cytotoxic activity of neither **1** nor **5** was affected by TQD, whereas co-treatment with TQD resulted in a dramatic 50-fold increase of the activity of docetaxel (Fig. 6a). Of note, TQD-induced p-gp blockade abolishes excretion of docetaxel (p-gp substrate) with an intracellular accumulation of the taxane resulting in cell death^41^. At the same time, co-treatment with TQD did not affect the activity of **1** or **5**, suggesting no relevant excretion of these molecules by p-gp (Fig. 6a). In line with this, the compounds **1** and **5** were equally active in both PC3 and DU145 (p-gp-low expressing) as well as in PC3-DR and DU145-DR (p-gp-high expressing) cells (Table 2), emphasizing a p-gp status-independent anticancer activity. Therefore, we hypothesized that compounds **1** and **5** similar to TQD are inhibitors of p-gp and are not p-gp substrates. Under this assumption, the diterpene compound **1** or **5** should also be able to increase docetaxel activity comparable to TQD (Fig. 6a). Due to the cytotoxic nature of the compounds **1** and **5**, we applied a Chou-Talalay method to evaluate their possible synergistic effect in combination with taxanes. Thus, we co-treated the drug-resistant PC3-DR and DU145-DR cells with docetaxel in combination with **1** (Fig. 6b) or in combination with **5** (Fig. 6c). Expectedly, the consecutive analysis of the data using SynergyFinder software revealed a pronounced synergistic effect of both combinations with docetaxel (Fig. 6b,c) suggesting an inhibition of p-gp. Of note, cabazitaxel reveals decreased affinity to p-gp and therefore is still active in patients suffering from docetaxel-resistant PCa. In line with this, combinational treatment with the isolated diterpenes **1** and **5** also resulted in the synergistic effect, however, this effect was expectedly less pronounced when compared to docetaxel (Fig. 6b,c).

## Conclusion

In conclusion, from the extract of the deep-water marine sponge *Spongionella* sp*.* we isolated one new compound called spongionellol A as well as five previously described compounds belonging to the family of spongian diterpenes. Two of these compounds, namely, spongionellol A (**1**) and 15,16-dideoxy-15α,17β-dihydroxy-15,17-oxidospongian-16-carboxylate 15,17-diacetate (**5**) exhibited high activity and selectivity in human PCa cells, independent of their resistance to currently available standard therapies. Cellular death was mainly caused by caspase-dependent apoptosis. Remarkably, both compounds were found to be potent inhibitors of p-gp. Hereby, the diterpenes were able to reverse the resistance to p-gp substrate docetaxel in the p-gp overexpressing taxane-resistant PC3-DR and DU145-DR cells resulting in synergistic cytotoxic effects of combinational treatment. Therefore, the isolated spongian diterpenes and similar compounds hold a promising potential as novel anticancer agents. They possess high activity and selectivity towards cancer cells combined with the ability to inhibit one of the major drug-resistance mediating mechanism of cancer cells.

## Supplementary Information

Supplementary Information.

## Data Availability

The data that support the findings of this study are available from the corresponding author upon reasonable request.
