# Supplementary material for: New diterpenes from the marine sponge Spongionella sp. overcome drug resistance in prostate cancer by inhibition of P-glycoprotein
Source: Sci Rep. 2022 Aug 9;12:13570. doi: 10.1038/s41598-022-17447-x (PMC9363487; doi:10.1038/s41598-022-17447-x)

Supporting Information

New Diterpenes from the Marine Sponge *Spongionella* sp. Overcome Drug Resistance in Prostate Cancer by Inhibition of P-Glycoprotein

Sergey A. Dyshlovoy[1,2,3,§,*], Larisa K. Shubina[4,§], Tatyana N. Makarieva[4], Jessica Hauschild[1,2], Nadja Strewinsky[1], Alla G. Guzii[4], Alexander S. Menshov[4], Roman S. Popov[4], Boris B. Grebnev[4], Tobias Busenbender[1], Su Jung Oh-Hohenhorst[2,5], Tobias Maurer[2,6], Derya Tilki[2,6], Markus Graefen[2], Carsten Bokemeyer[1], Valentin A. Stonik[4,‡], Gunhild von Amsberg[1,2,‡]

1 Department of Oncology, Hematology and Bone Marrow Transplantation with Section Pneumology, Hubertus Wald Tumorzentrum – University Cancer Center Hamburg (UCCH), University Medical Center Hamburg-Eppendorf, Hamburg, Germany

2 Martini-Klinik, Prostate Cancer Center, University Hospital Hamburg-Eppendorf, Hamburg, Germany

3 Institute of Science-Intensive Technologies and Advanced Materials, Far Eastern Federal University, Vladivostok, Russian Federation

4 G.B. Elyakov Pacific Institute of Bioorganic Chemistry, Far-East Branch, Russian Academy of Sciences, Vladivostok, Russian Federation

5 Centre de recherche du Centre hospitalier de l'Université de Montréal (CRCHUM) et Institut du cancer de Montréal, Montréal, Quebec, Canada

6 Department of Urology, University Hospital Hamburg-Eppendorf, Hamburg, Germany

§ Equal first authorship

‡ Equal last authorship

***** Correspondence: Sergey A. Dyshlovoy; e-mail: s.dyshlovoy@uke.de

Table S1. List of antibodies used.

Antibodies	Clonality	Source	Cat.-No.	Dilution	Manufacturer
anti-AR	pAb	rabbit	sc-816	1:200	Santa Cruz
anti-AR-V7	mAb	rabbit	198394	1:1000	abcam
anti-cleaved caspase-3	mAb	rabbit	#9664	1:1000	Cell Signaling
anti-PARP	pAb	rabbit	#9542	1:1000	Cell Signaling
anti-PSA/KLK3	mAb	rabbit	#5365	1:1000	Cell Signaling
anti-survivin	pAb	rabbit	NB500-201	1:1000	Novus
anti-LC3B-I/II	pAb	rabbit	#2775	1:1000	Cell Signaling
anti-MDR1 (p-gp)	mAb	rabbit	#13342	1:1000	Cell Signaling
anti-rabbit IgG-HRP		goat	#7074	1:5000	Cell Signaling
anti-mouse IgG-HRP		sheep	NXA931	1:10000	GE Healthcare
anti-α-Tubulin	mAb	mouse	T5168	1:5000	Sigma-Aldrich
anti-β-Actin-HRP	pAb	goat	sc-1616	1:10000	Santa Cruz

Figure S1. ^{1}H NMR spectrum of spongionellol A (**1**) in $CDCl_3$.

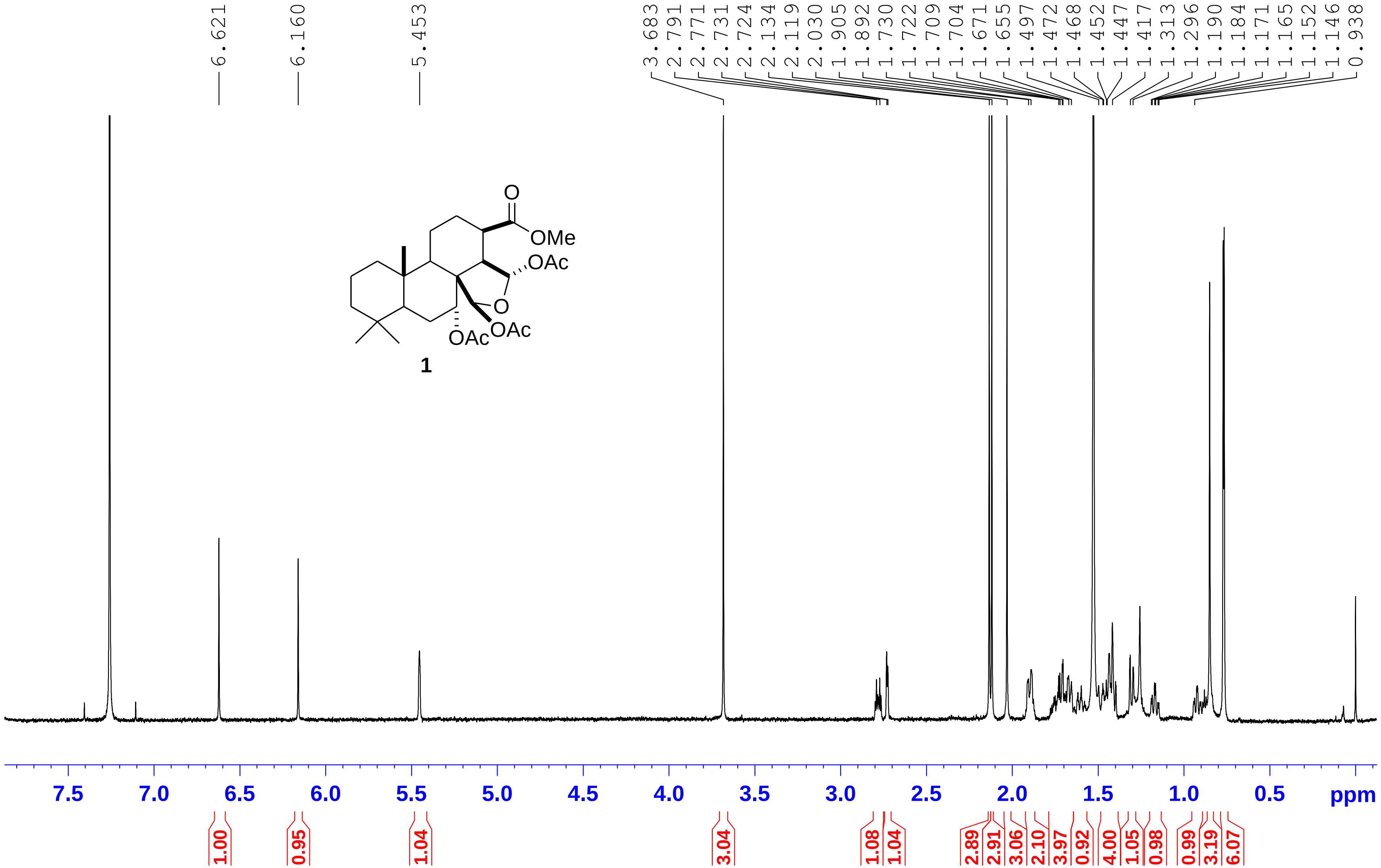

Figure S2. ^{13}C NMR spectrum of spongionellol A (**1**) in $CDCl_3$.

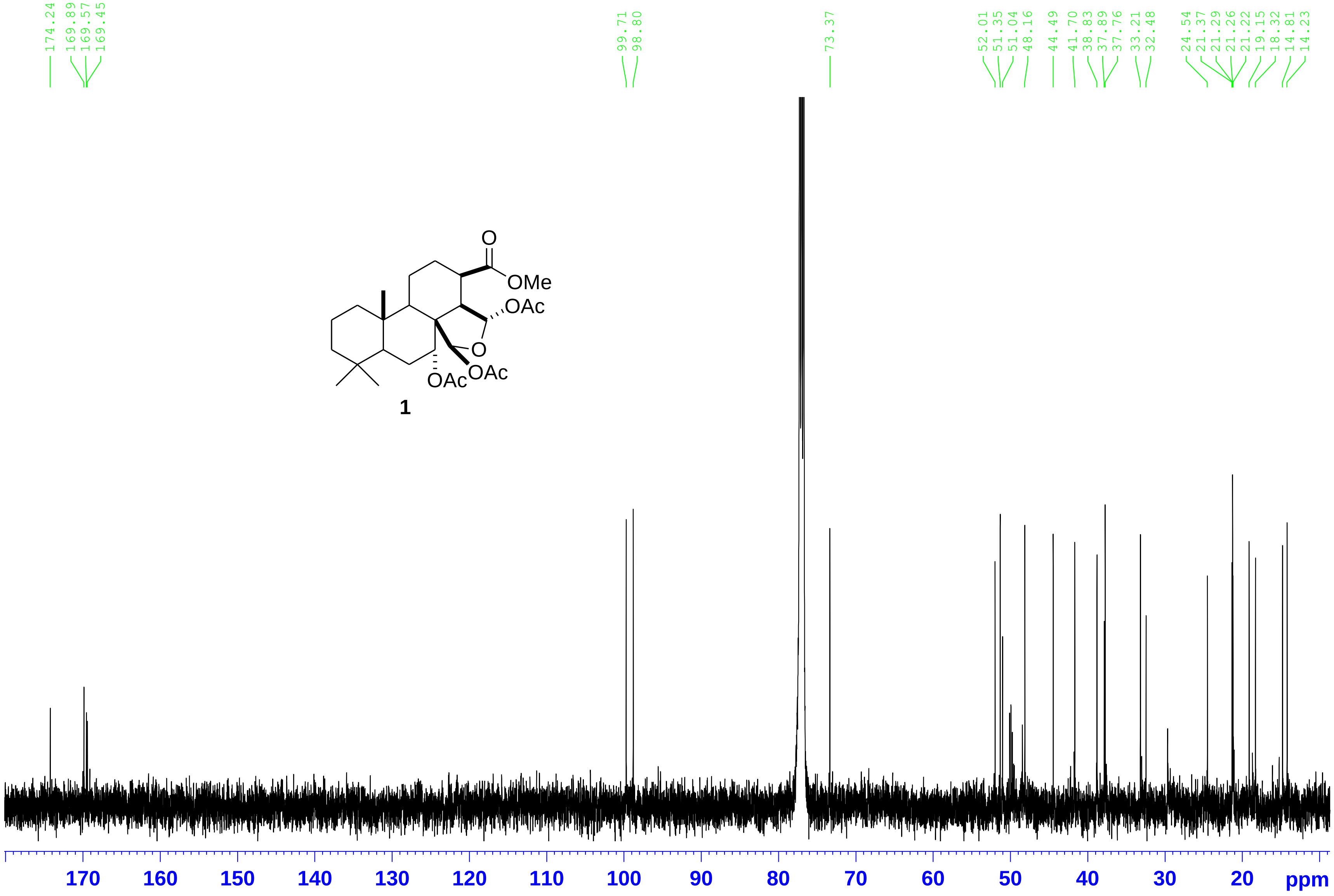

Figure S3. ^1H-^1H-COSY spectrum of spongionellol A (**1**) in $CDCl_3$.

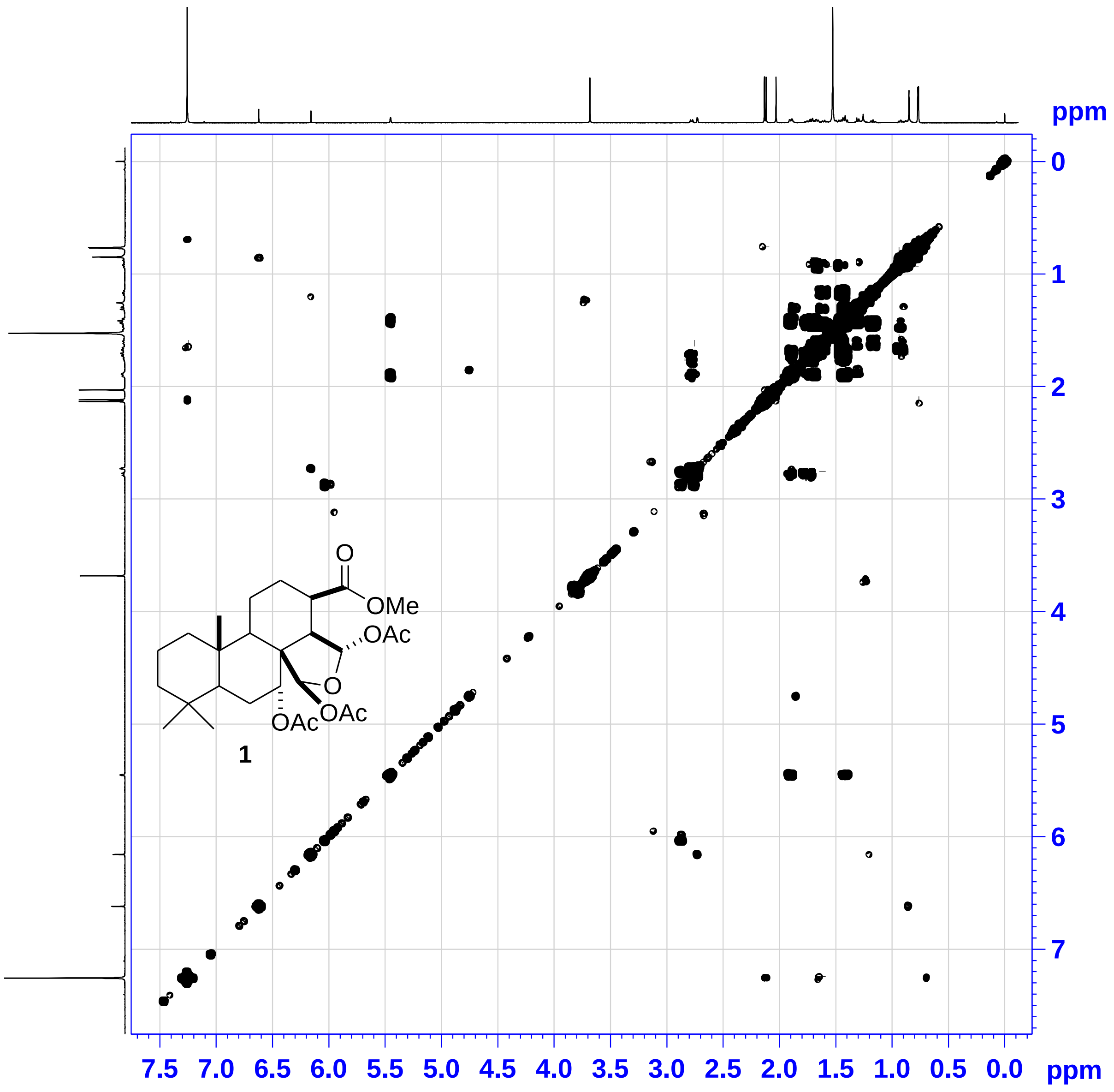

Figure S4. HSQC spectrum of spongionellol A (**1**) in $CDCl_3$.

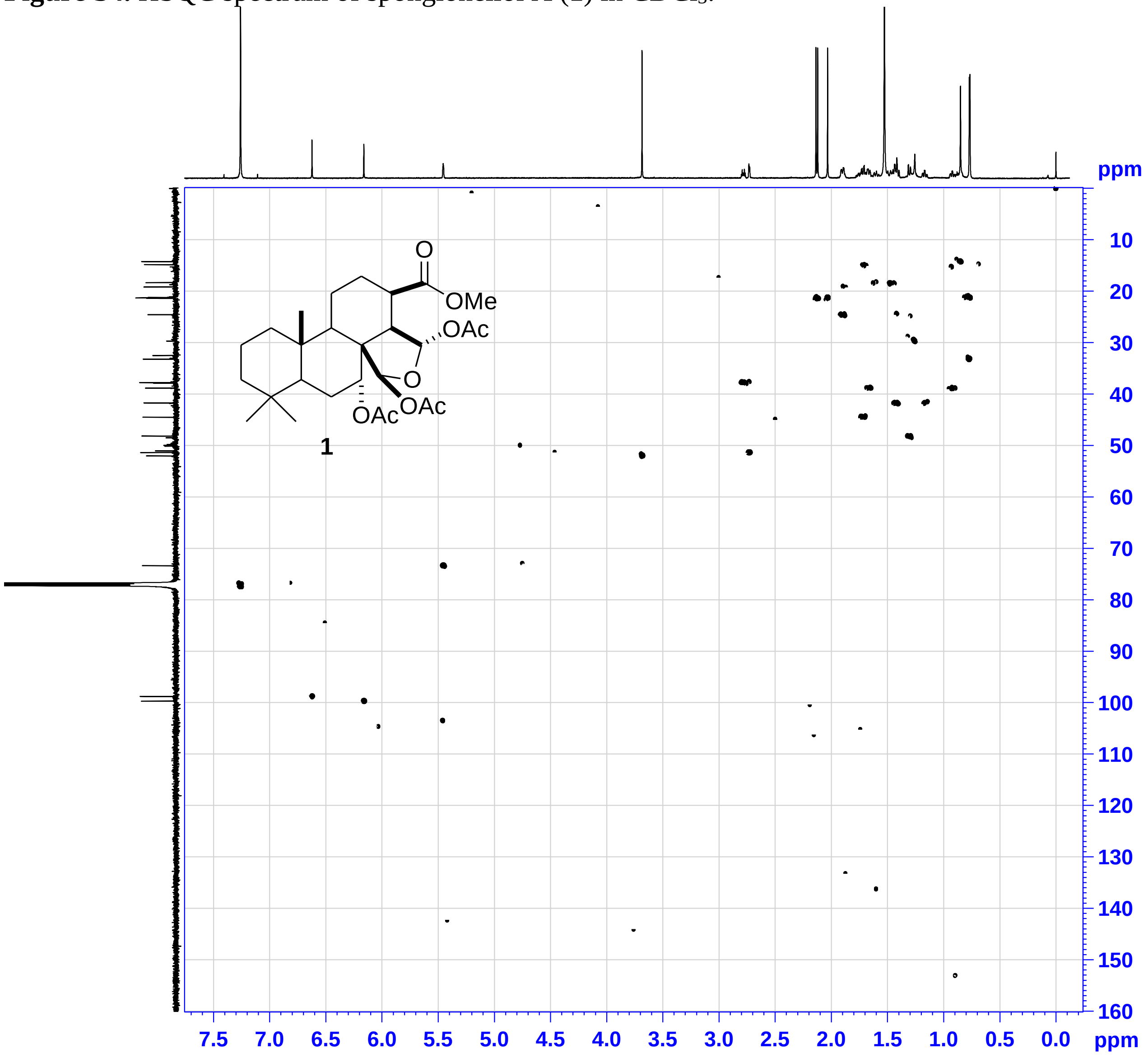

Figure S5. HMBC spectrum of spongionellol A (**1**) in $CDCl_3$.

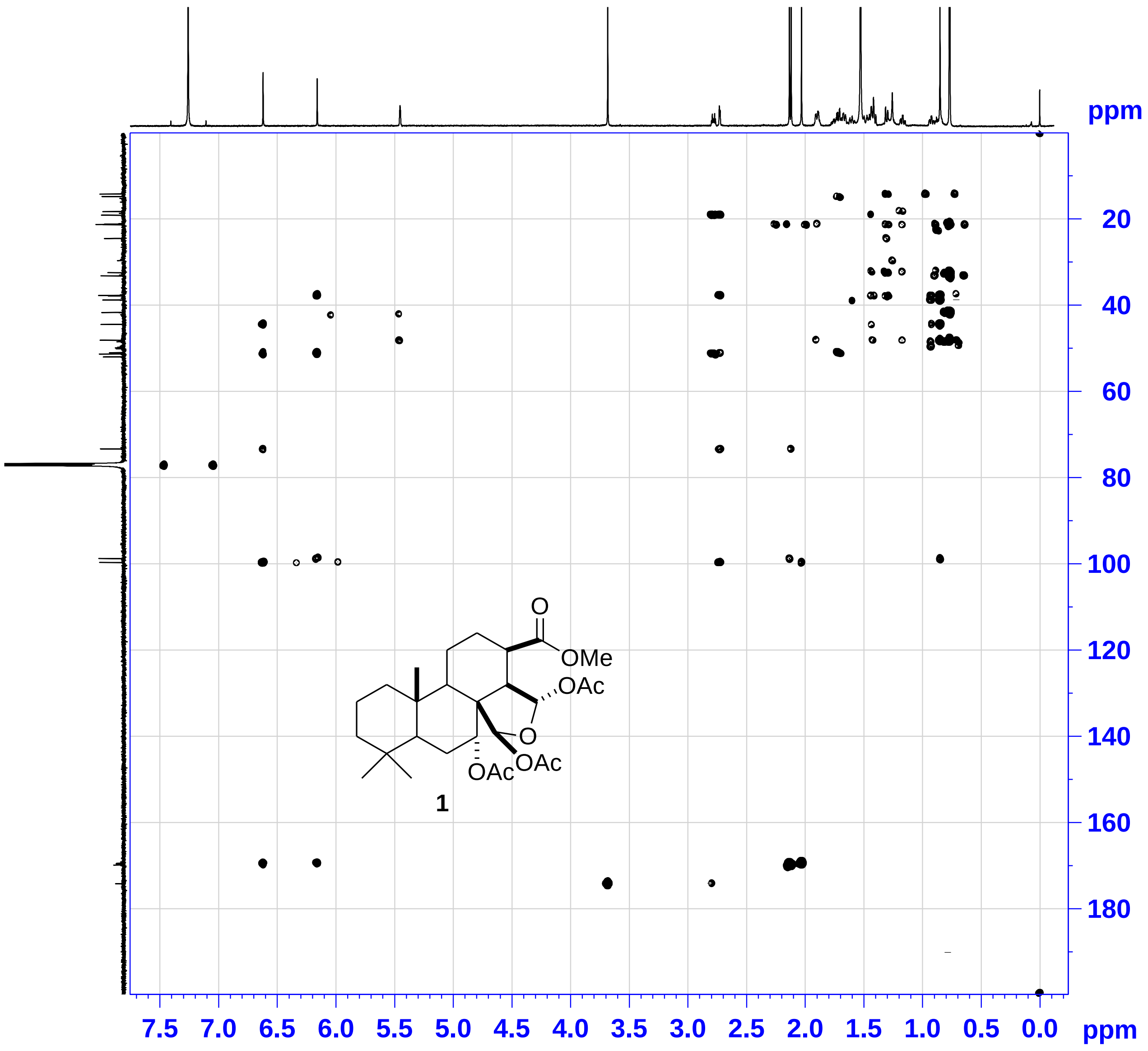

Figure S6. NOESY spectrum of spongionellol A (**1**) in $CDCl_3$.

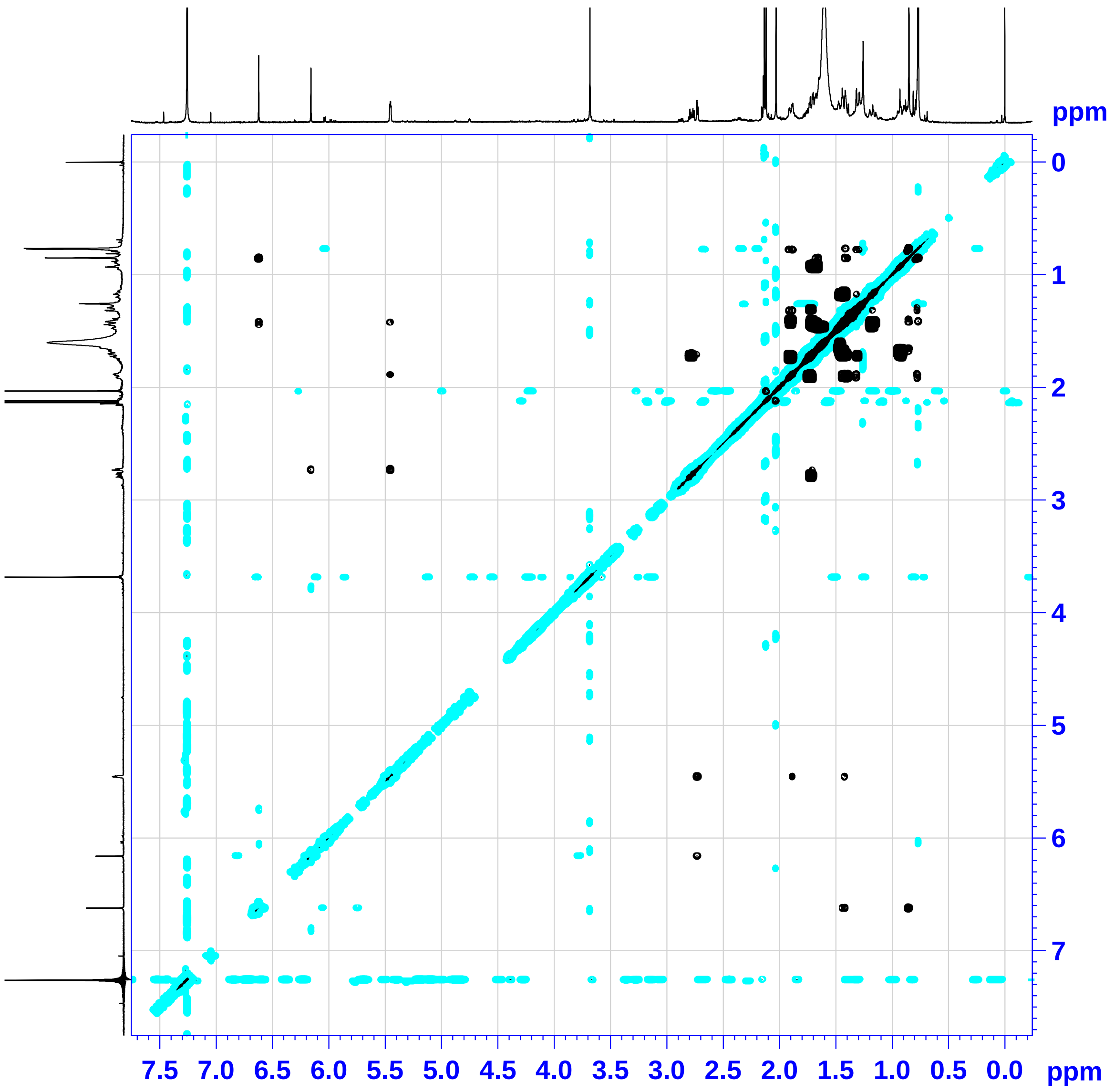

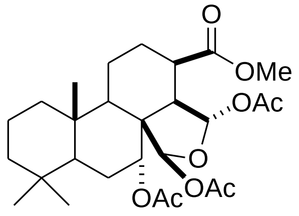

1

Figure S7. The HRESIMS and MS/MS spectra of compound **1**.

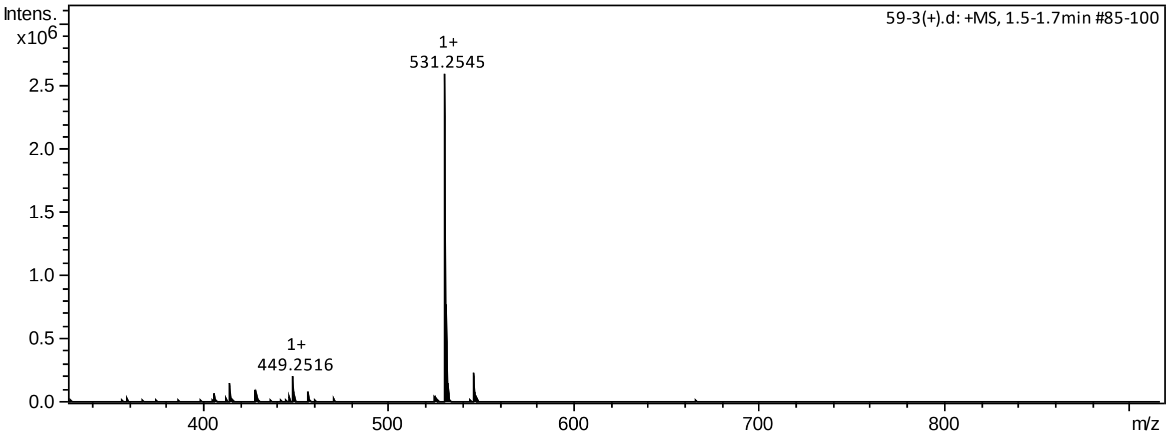

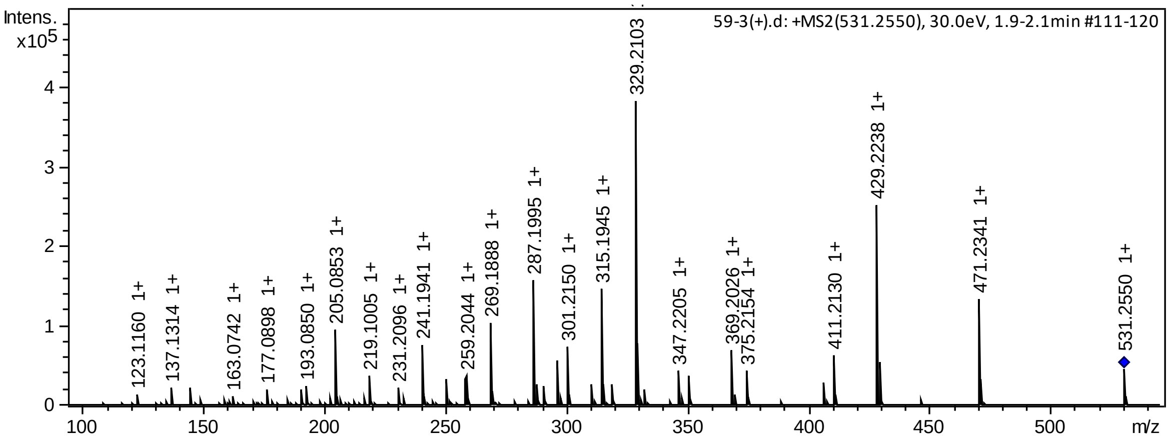

Figure S8. ^{1}H NMR spectrum of compound **2** in $CDCl_3$.

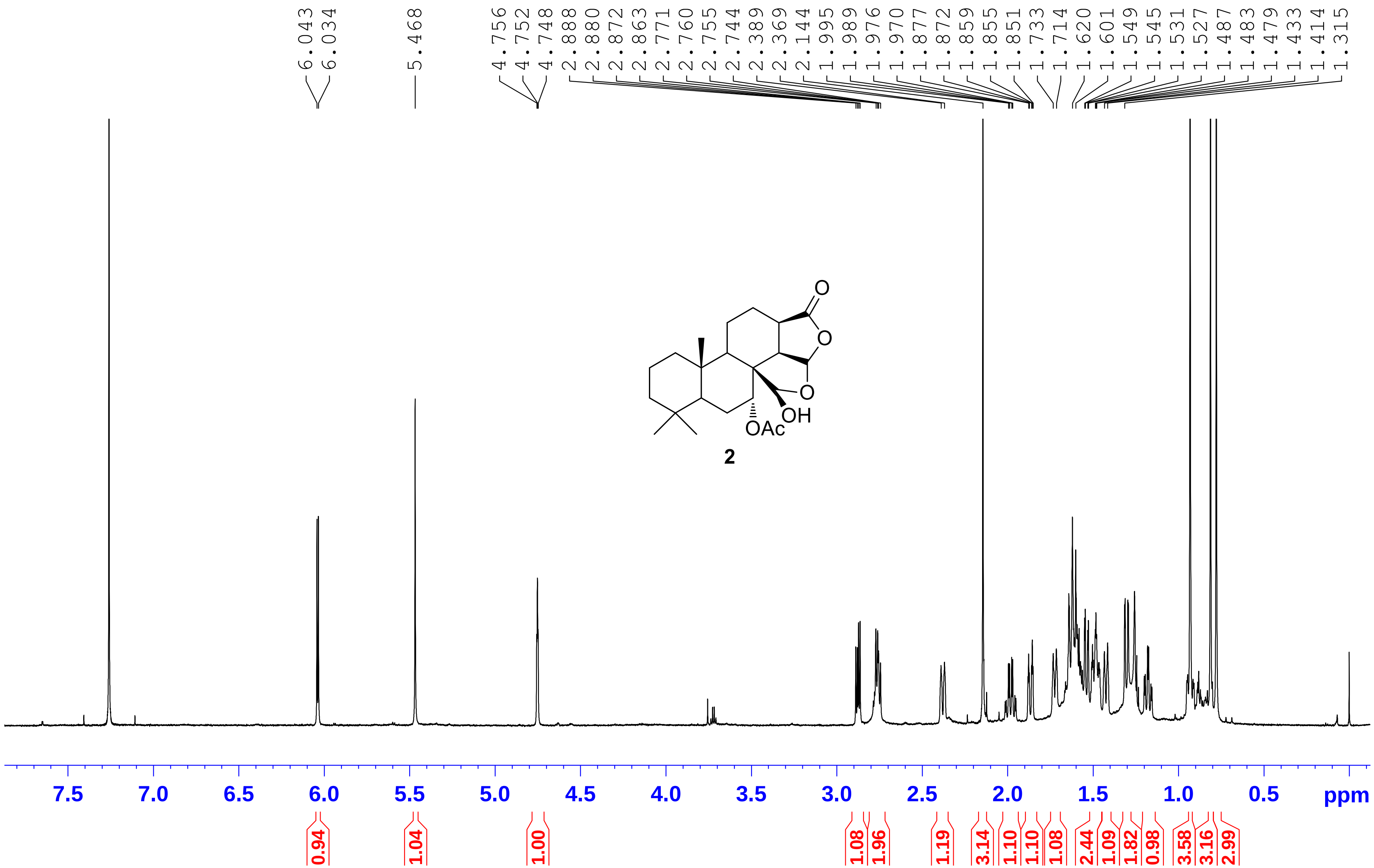

Figure S9. ^{13}C NMR spectrum of compound **2** in $CDCl_3$.

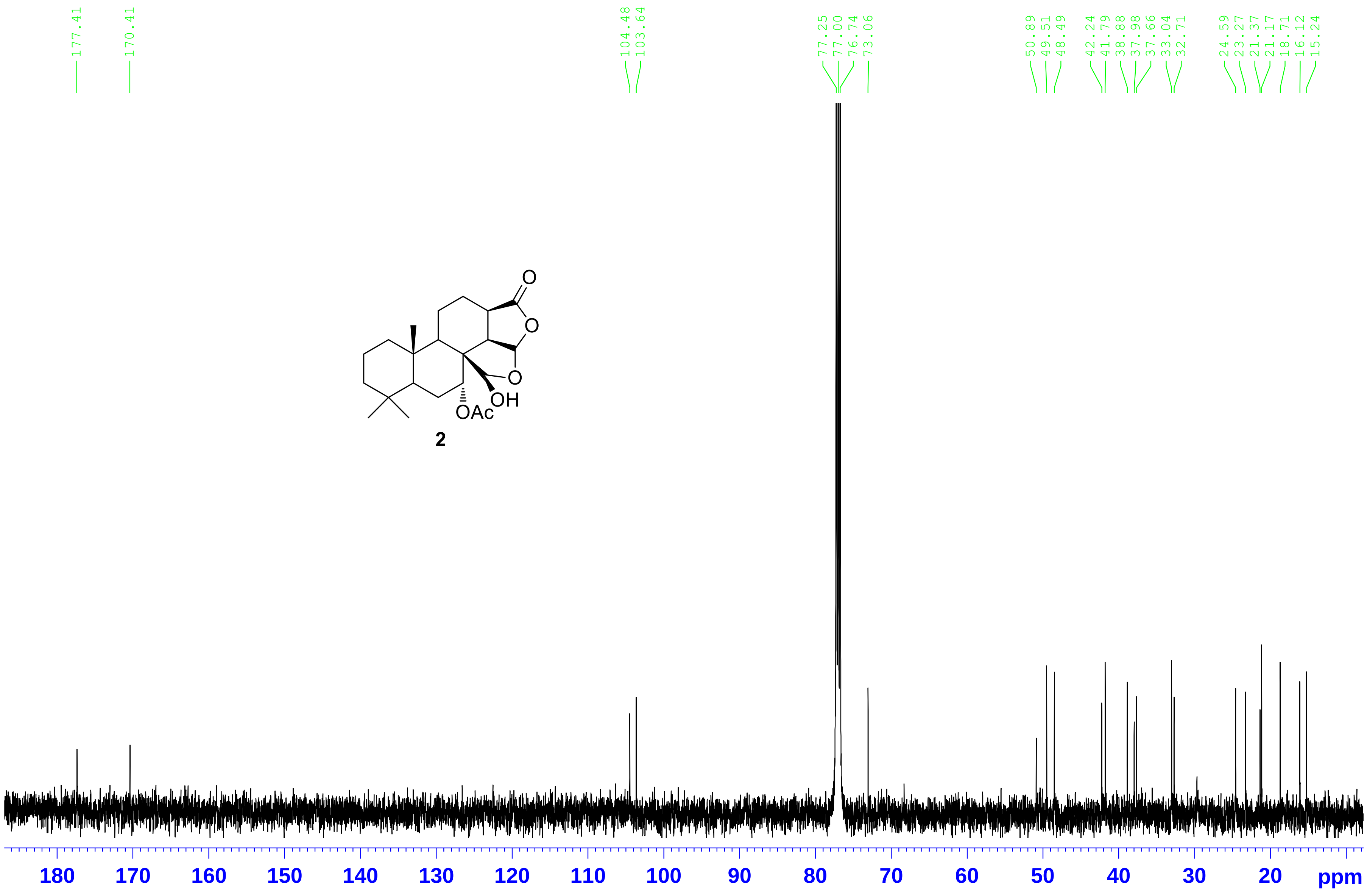

Figure S10. ^{1}H NMR spectrum of compound **3** in $CDCl_3$.

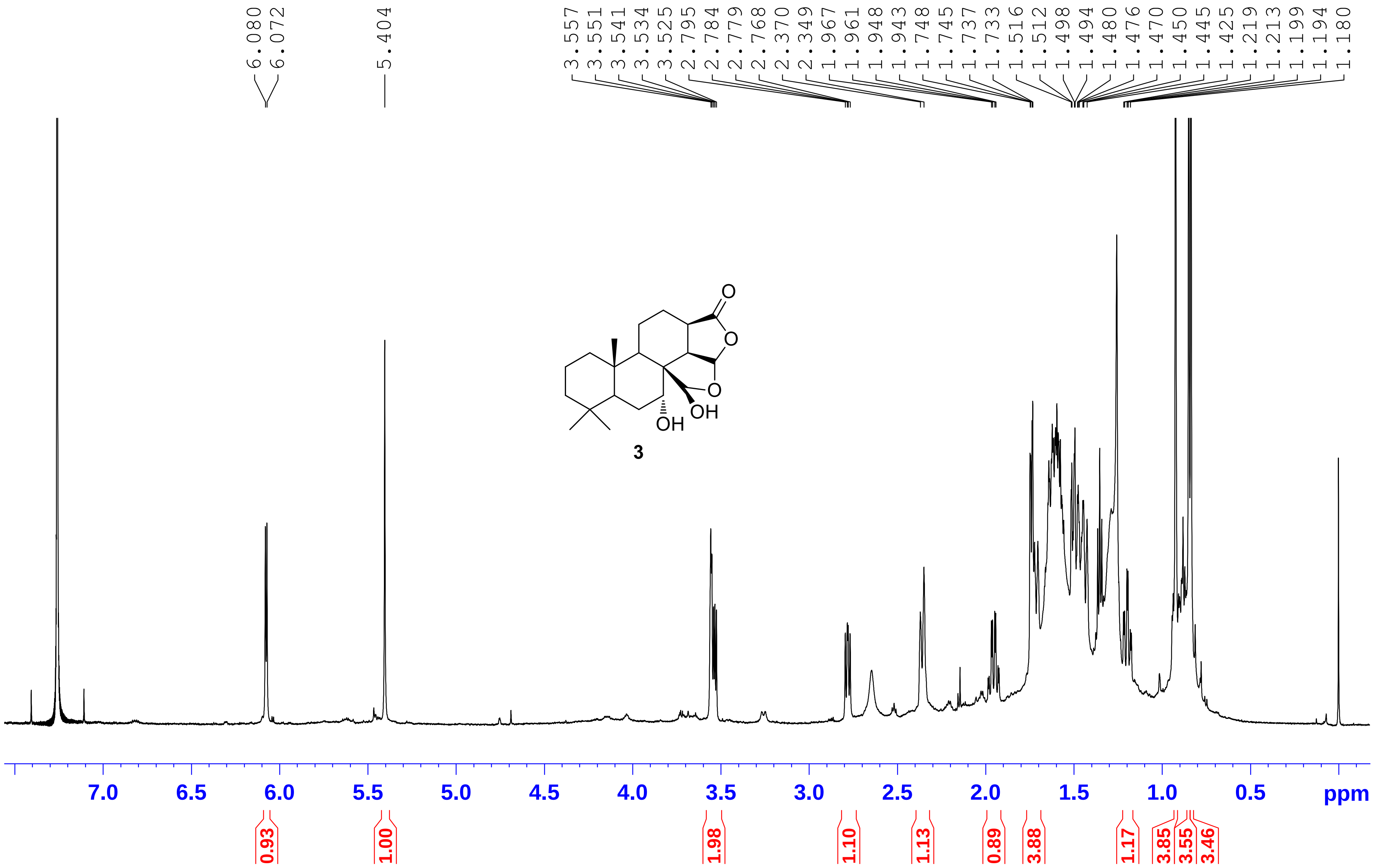

Figure S11. ^{13}C NMR spectrum of compound **3** in $CDCl_3$.

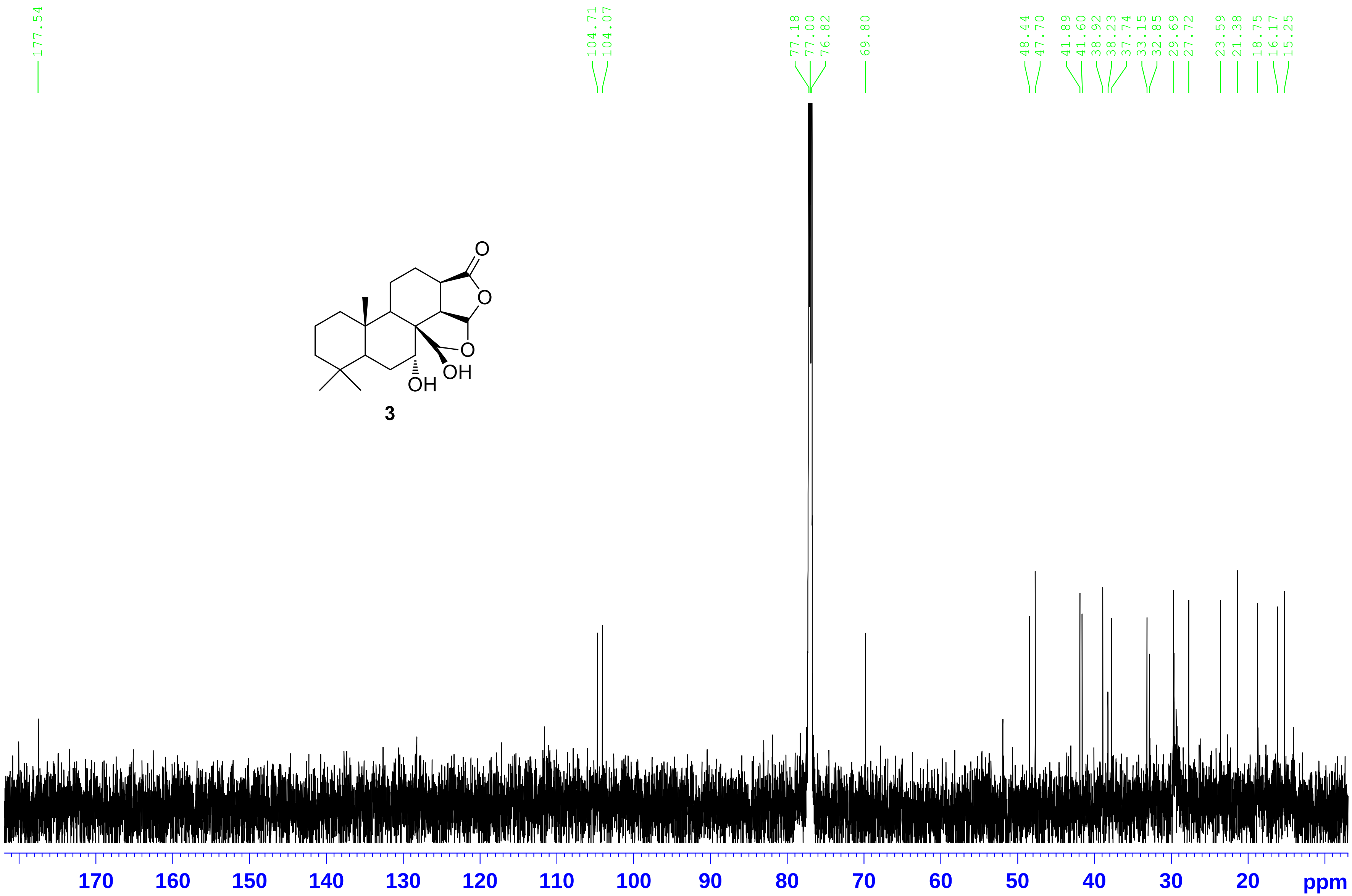

Figure S12. ^{1}H NMR spectrum of compound **4** in $CDCl_3$.

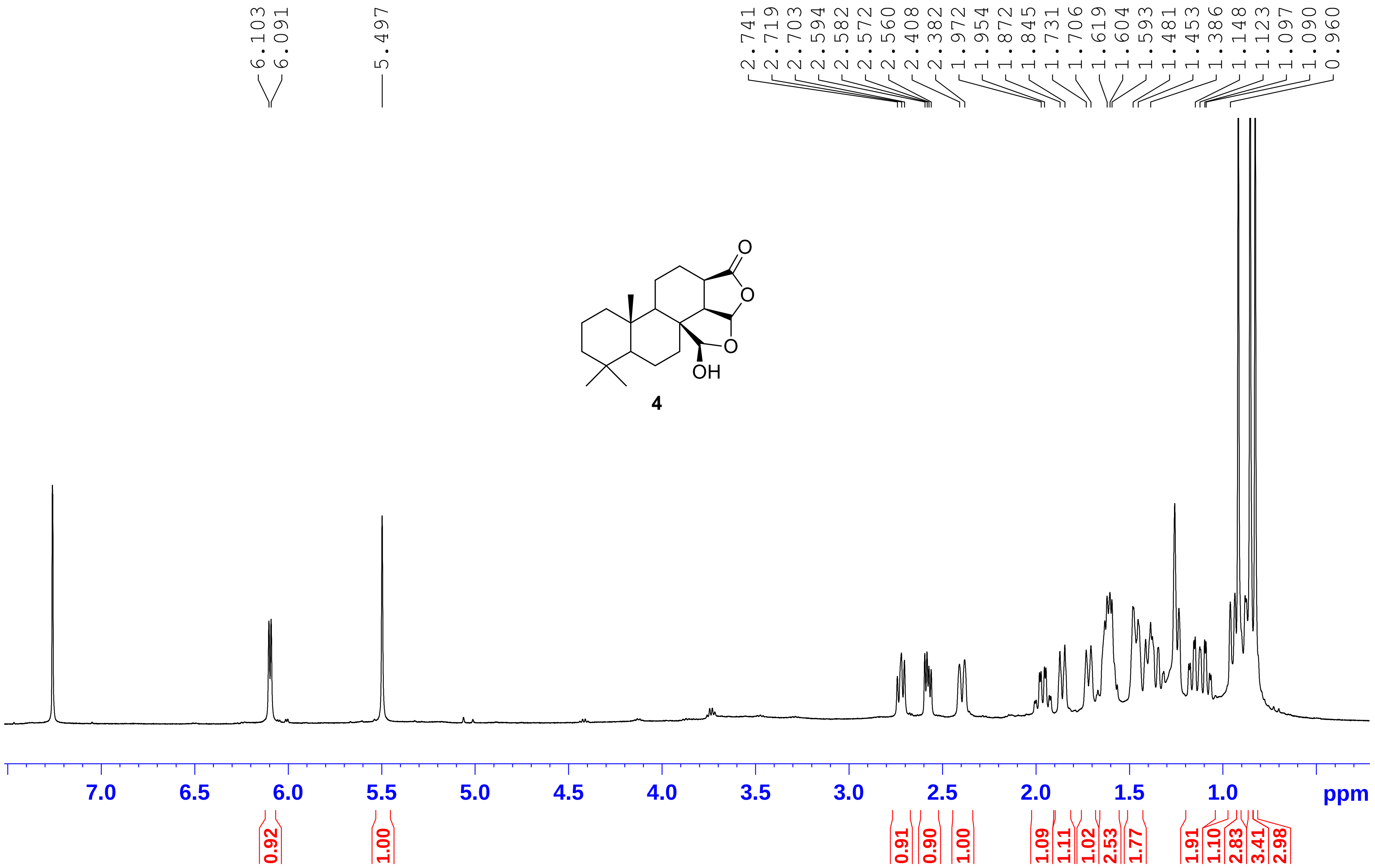

Figure S13. ^{13}C NMR spectrum of compound **4** in $CDCl_3$.

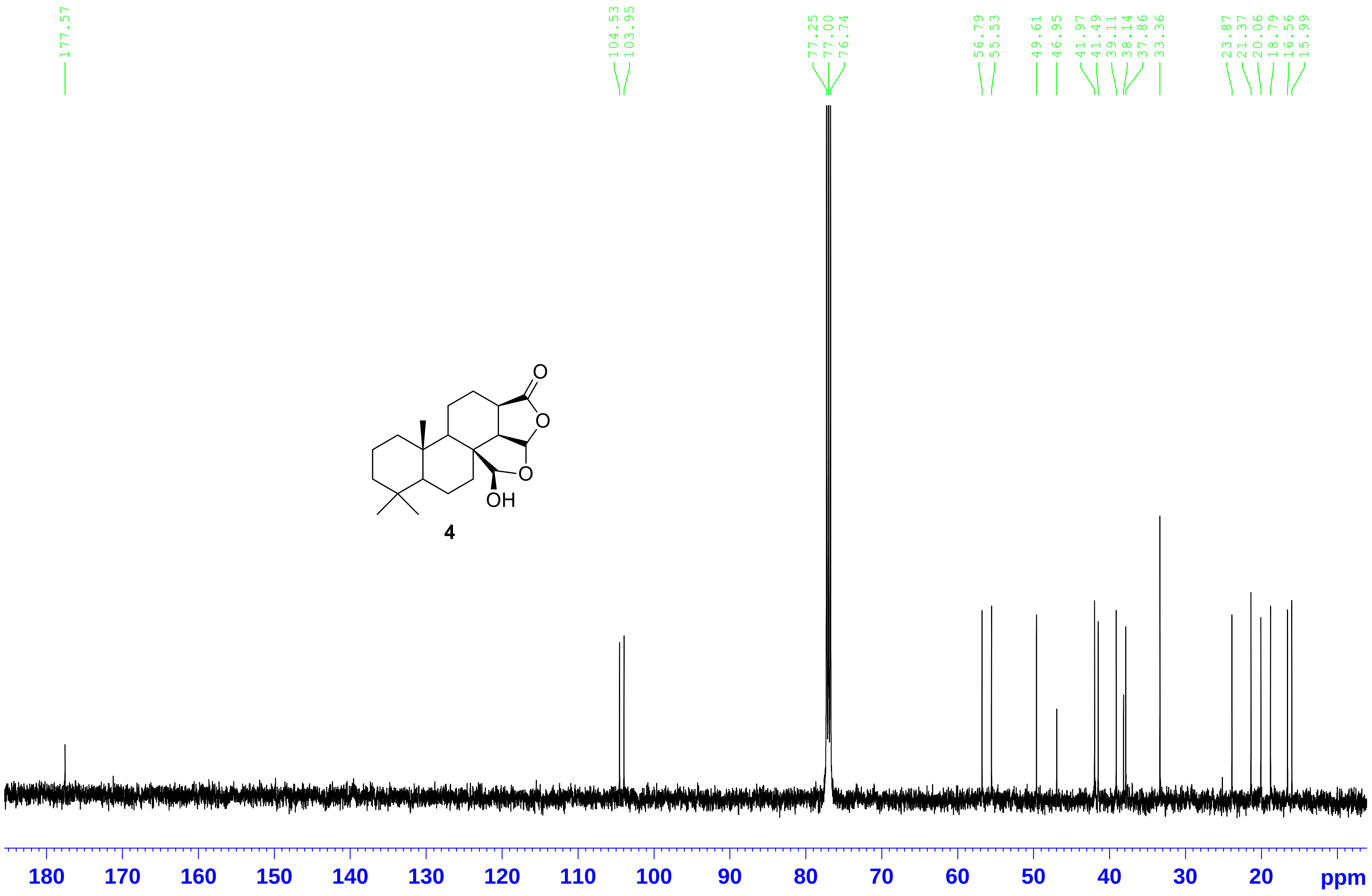

Figure S14. ^{1}H NMR spectrum of compound **5** in $CDCl_3$.

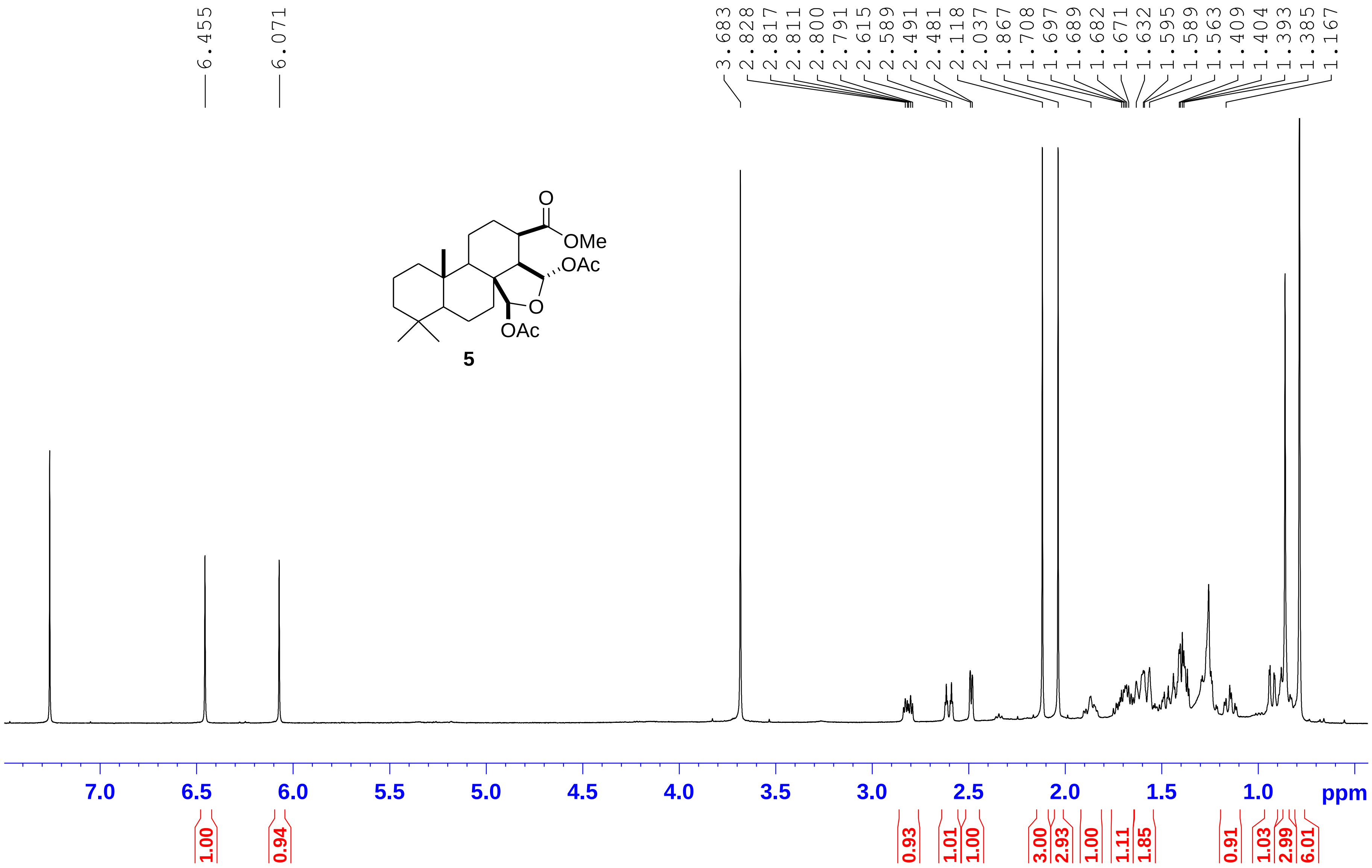

Figure S15. ^{13}C NMR spectrum of compound **5** in $CDCl_3$.

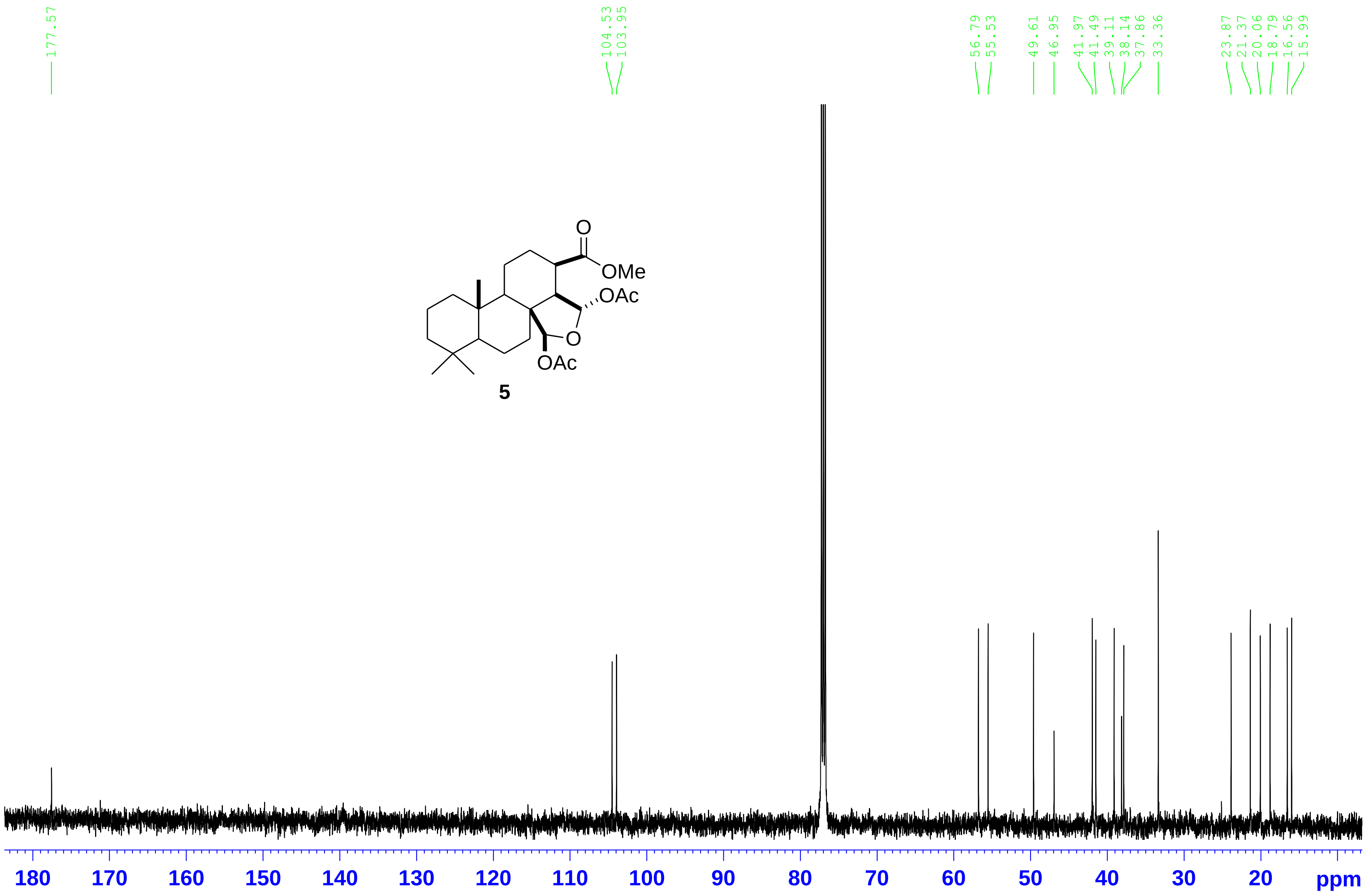

Figure S16. ^{1}H NMR spectrum of compound **6** in $CDCl_3$.

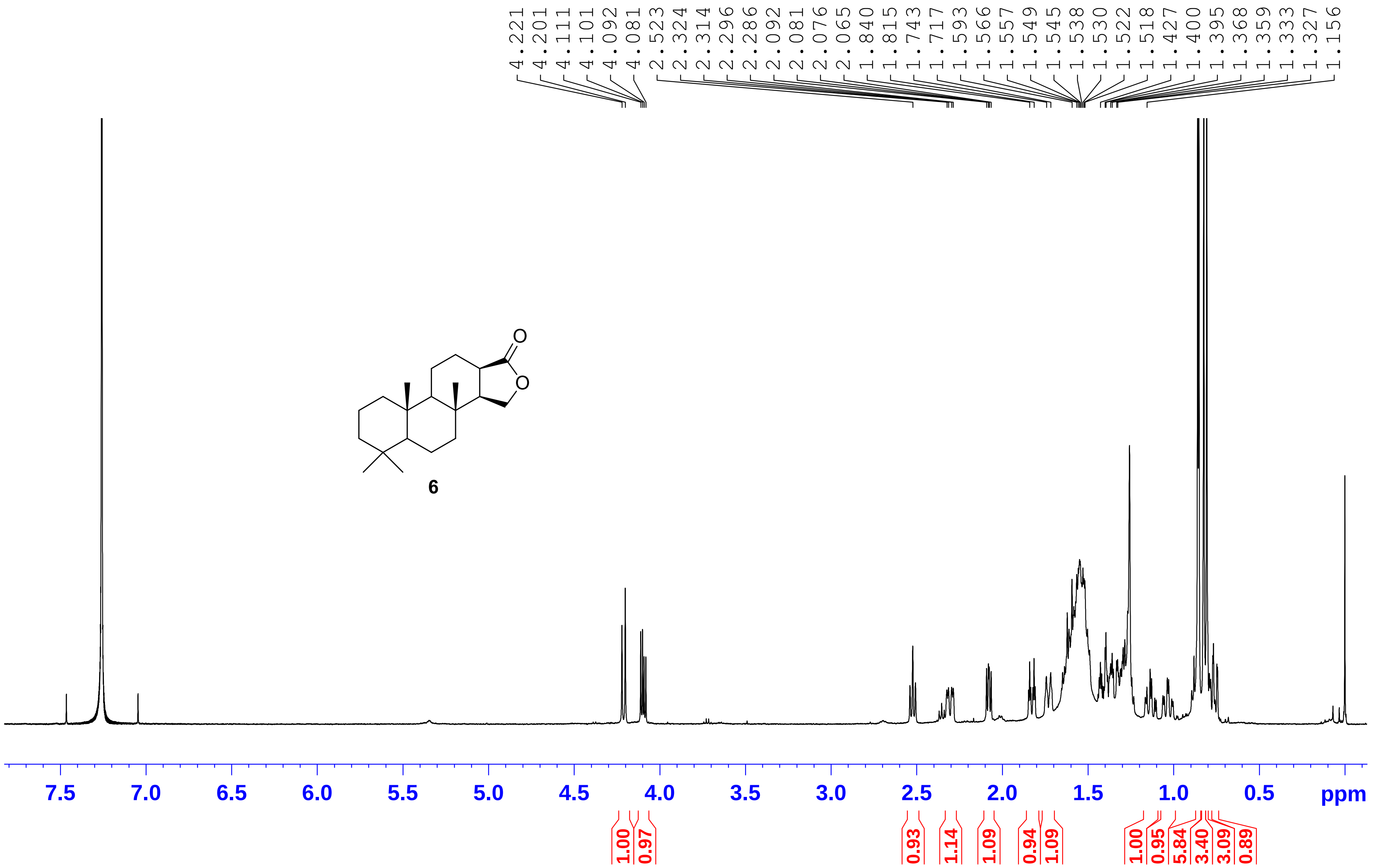

Figure S17. ^{13}C NMR spectrum of compound **6** in $CDCl_3$.

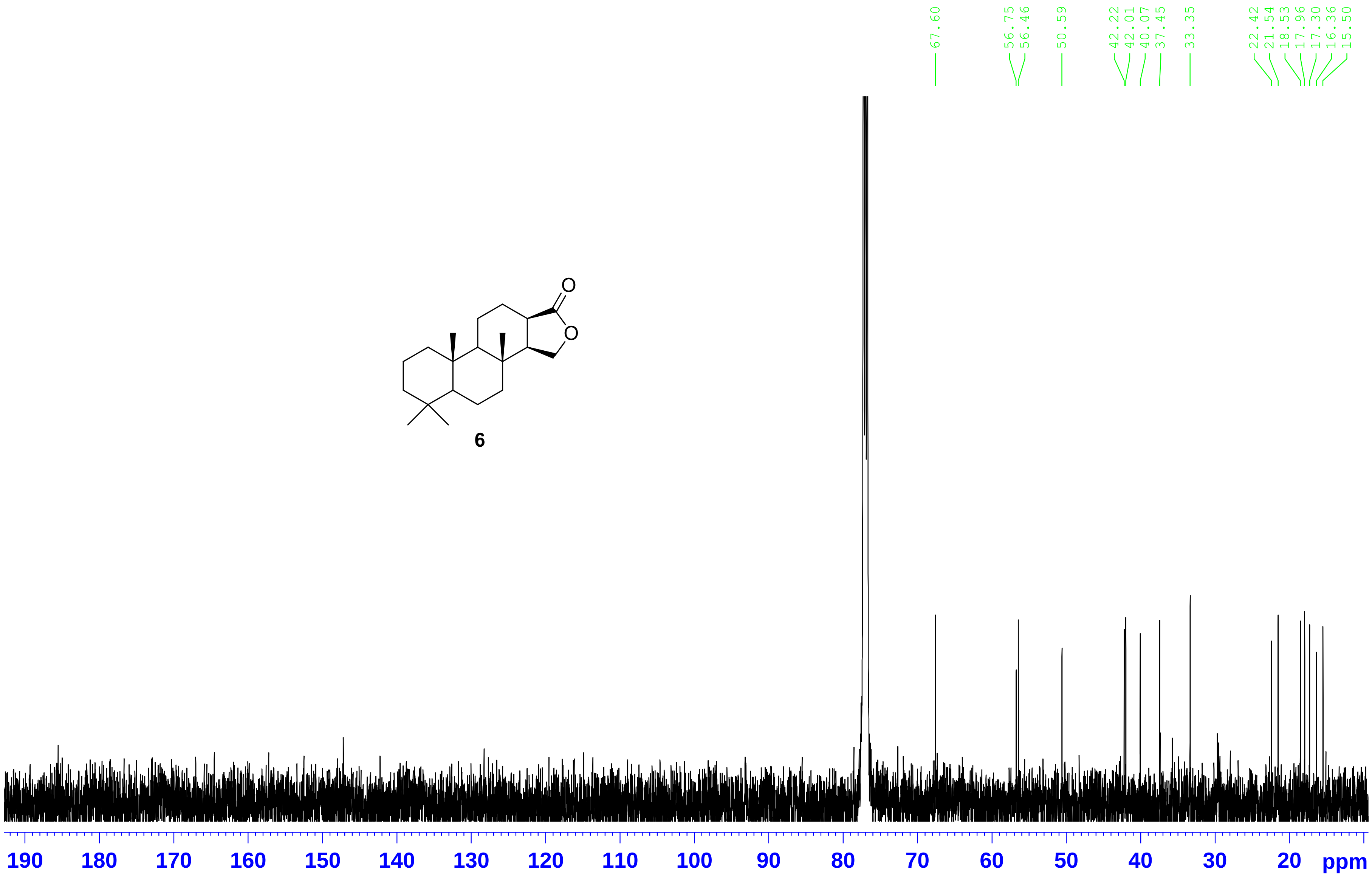

Figure S18. The original full-size blots corresponding to the data represented on Fig. 3a. Red box and arow indicates relevant bands represented in the Manuscript.

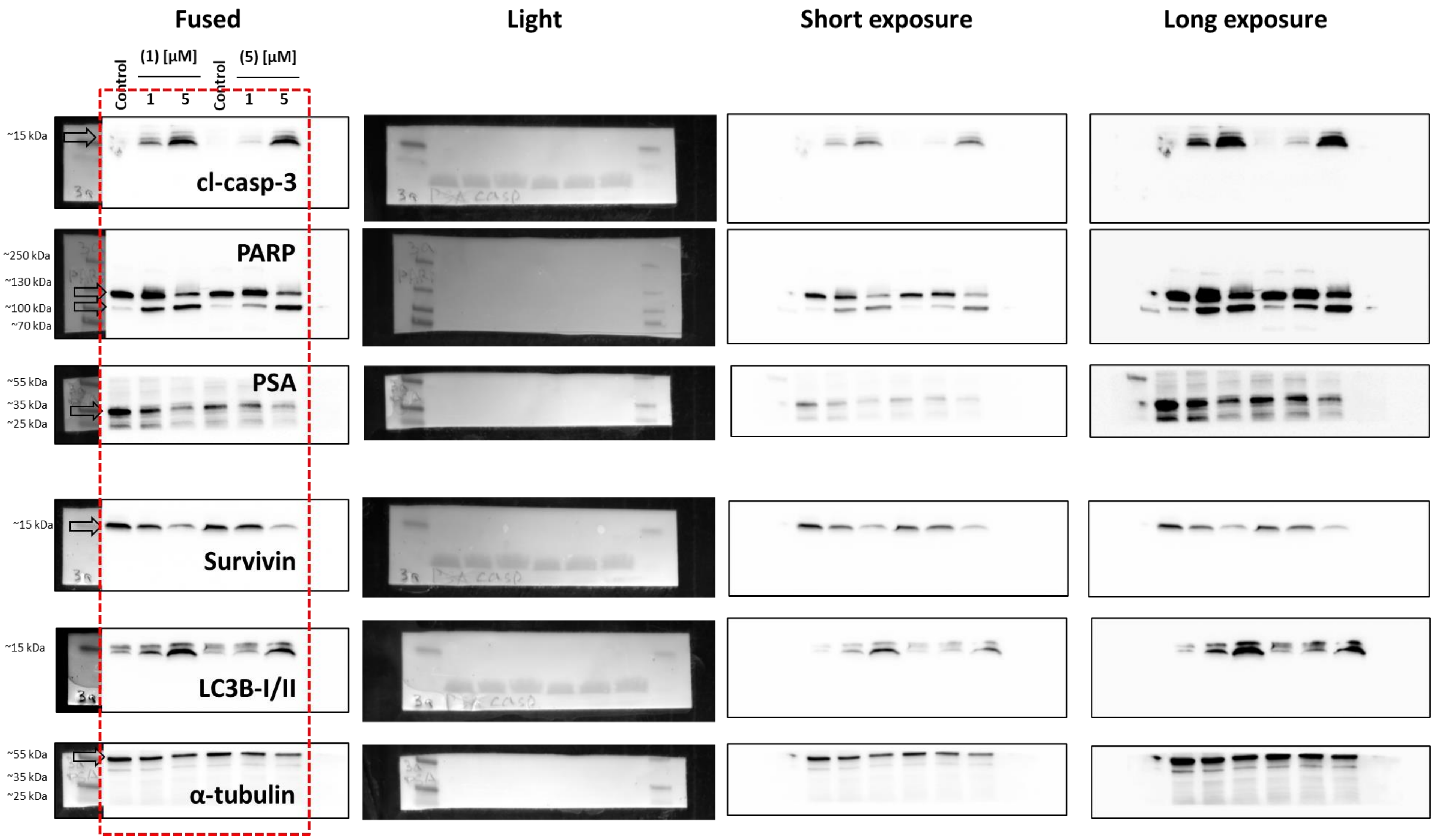

Figure S18 (continuation). The original full-size blots corresponding to the data represented on Fig. 3a. Red box and arow indicates relevant bands represented in the Manuscript.

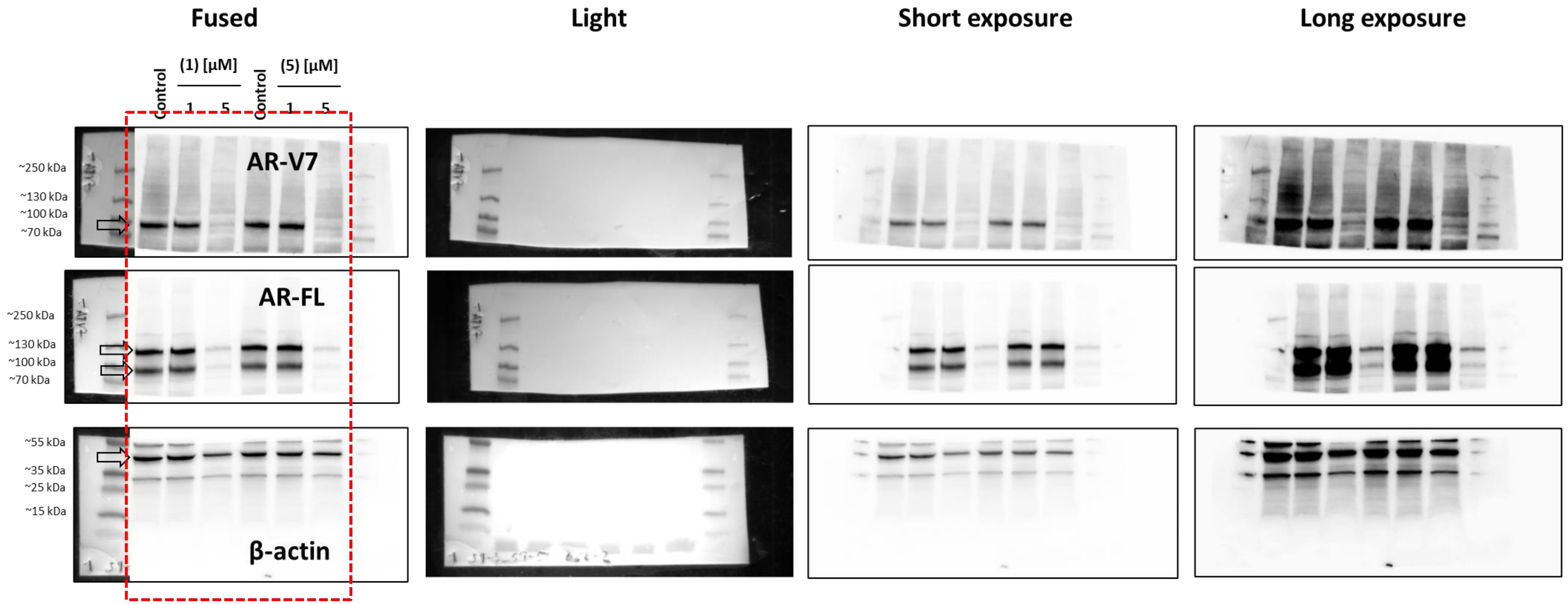

Figure S19. The original full-size blots corresponding to the data represented on Fig. 4a. Red box and arow indicates relevant bands represented in the Manuscript.

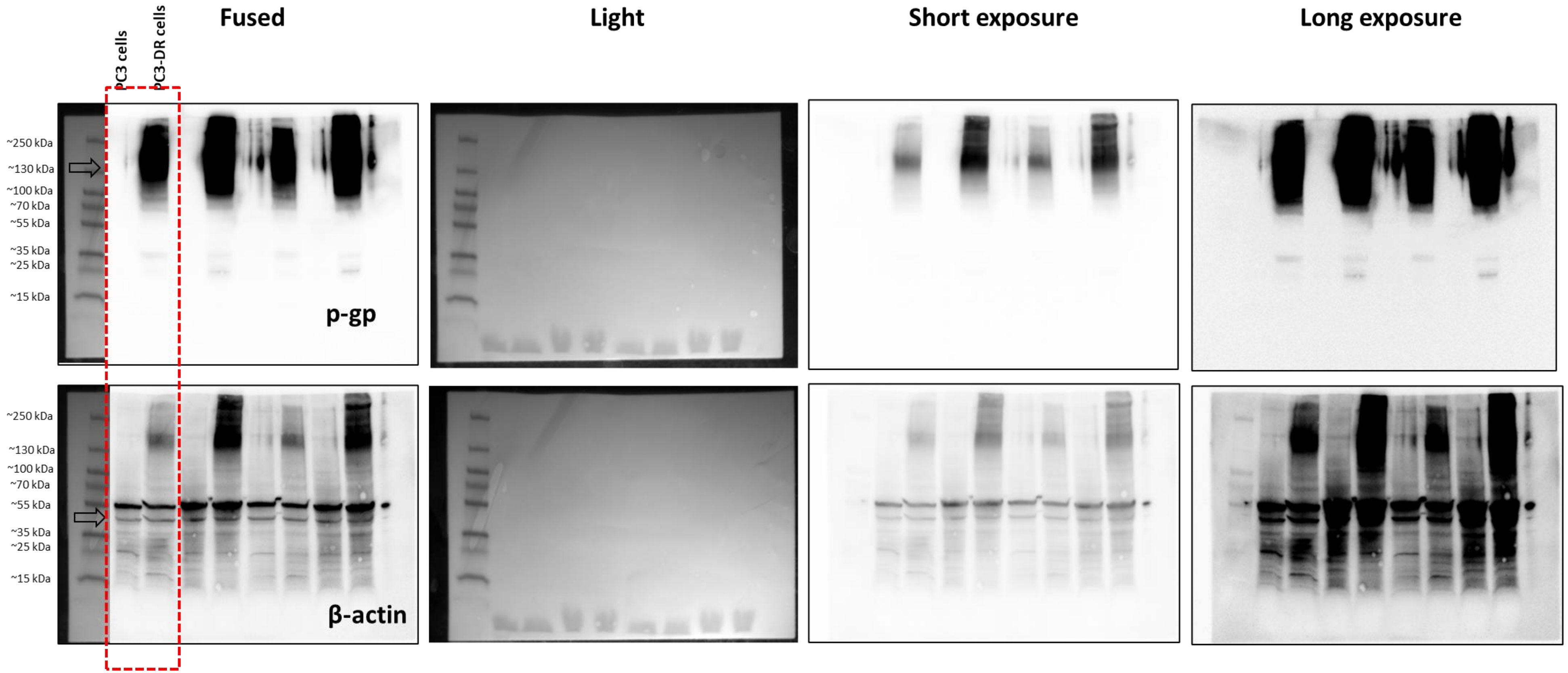

Figure S19 (continuation). The original full-size blots corresponding to the data represented on Fig. 4a. Red box and arow indicates relevant bands represented in the Manuscript.

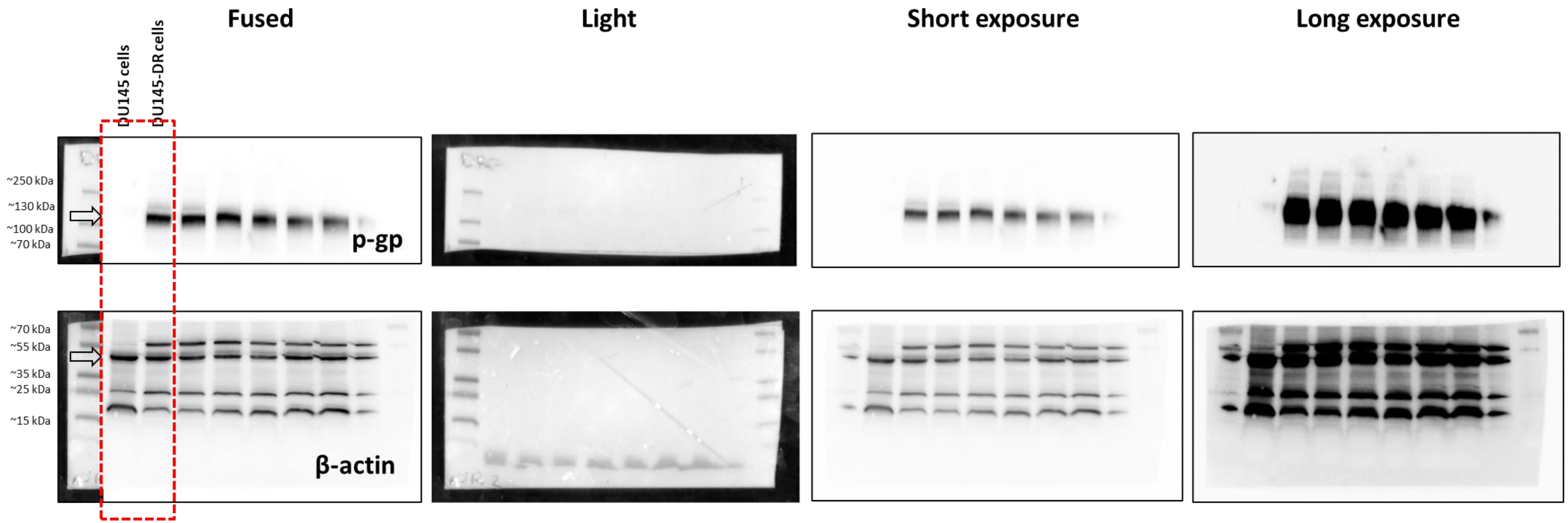

Figure S20. The original full-size blots corresponding to the data represented on Fig. 5a. Red box and arow indicates relevant bands represented in the Manuscript.

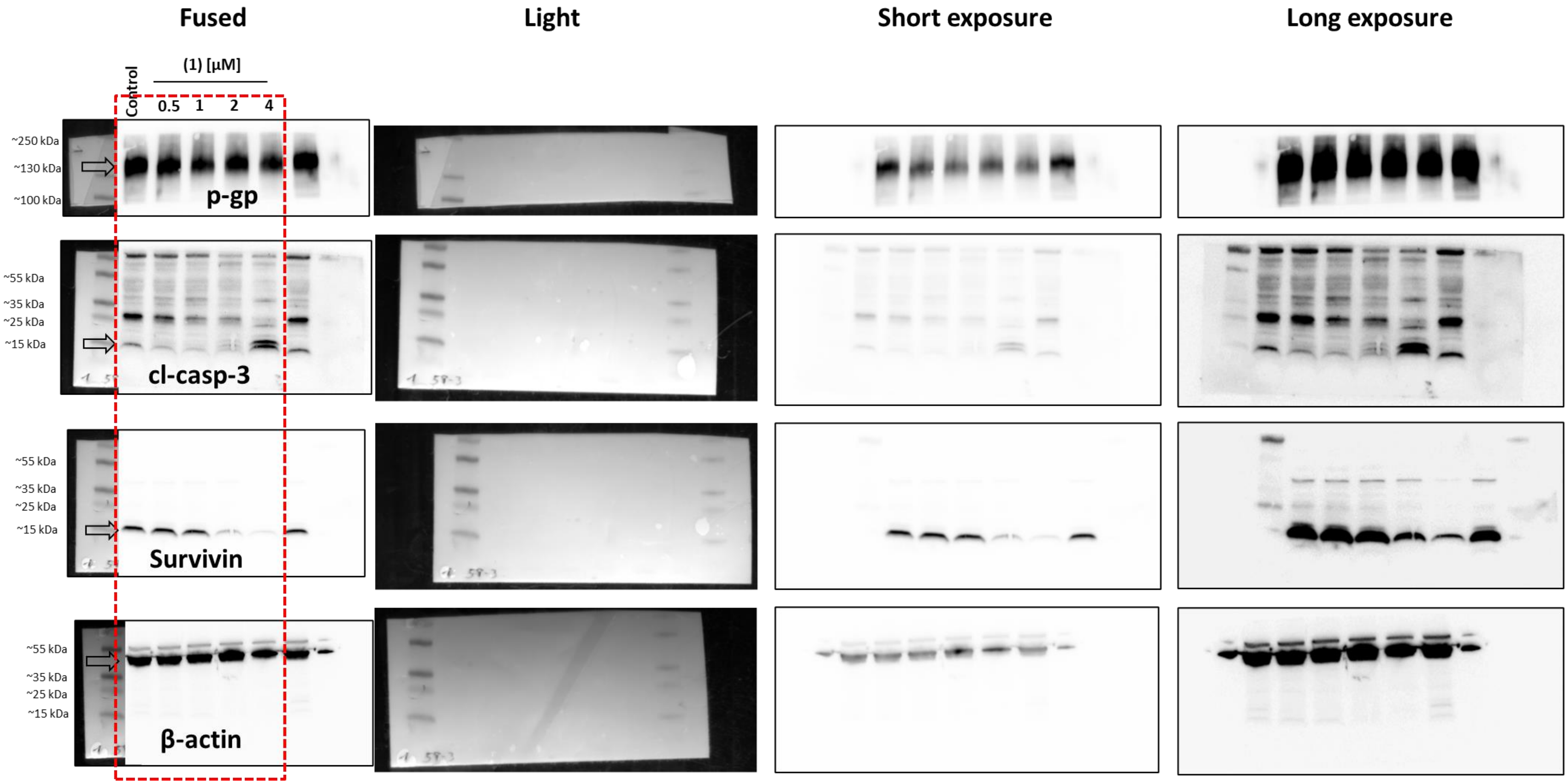

Figure S20 (continuation). The original full-size blots corresponding to the data represented on Fig. 5a. Red box and arow indicates relevant bands represented in the Manuscript.

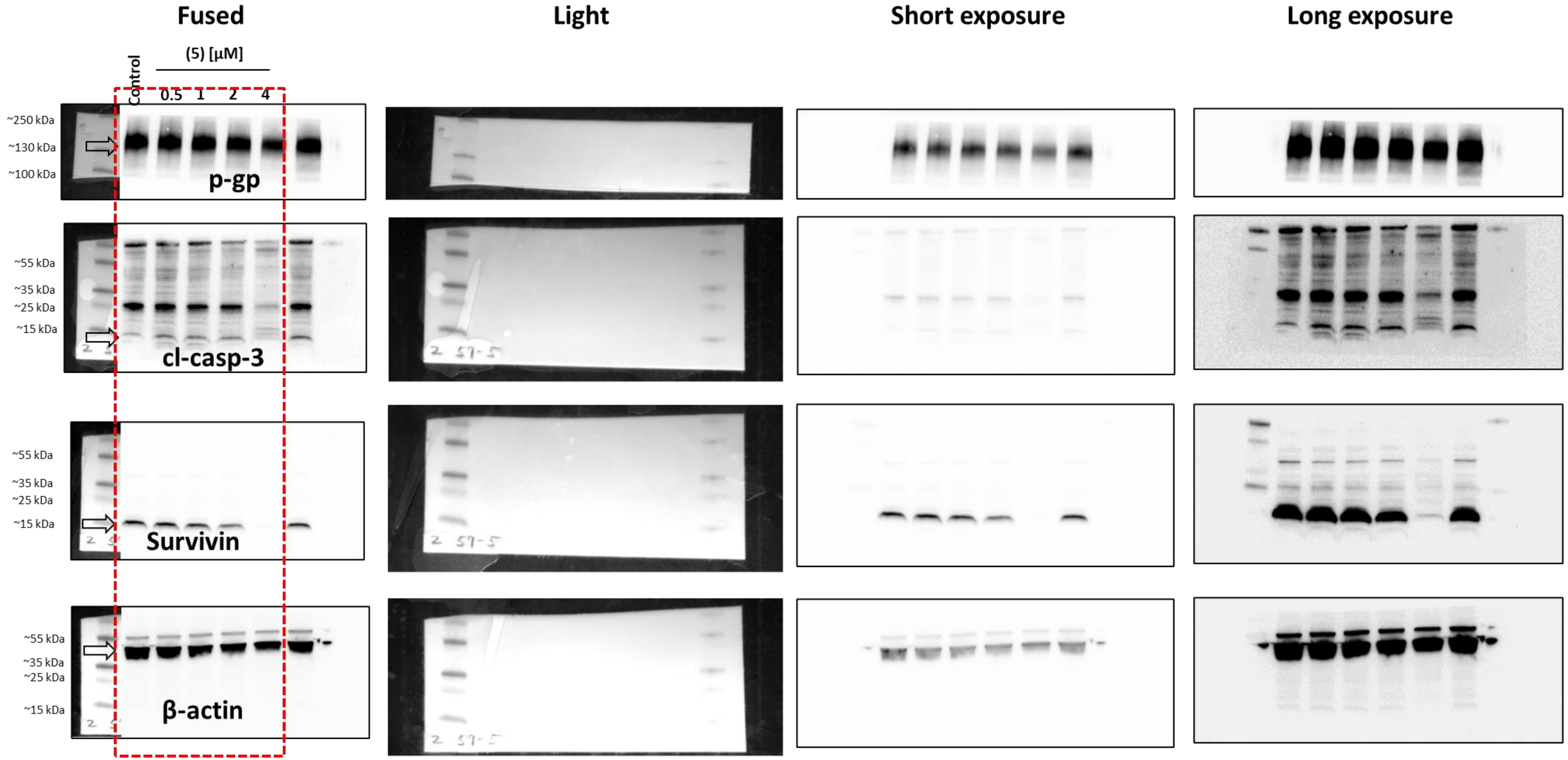

Supplement: Supplementary file 1 — Supplementary Information. [file 41598_2022_17447_MOESM1_ESM.pdf]
